# Cold‐Inducible RNA‐Binding Protein Alleviates Hypertensive Renal Injury Through the KEAP1‐NRF2 Pathway

**DOI:** 10.1002/smmd.70062

**Published:** 2026-11-07

**Authors:** Yue Zhang, Qilin Li, Man Luo, Dongxu Hua, Yanhui Sheng, Lei Wang, Peng Li

**Affiliations:** ^1^ Department of Cardiology The First Affiliated Hospital with Nanjing Medical University Nanjing China; ^2^ Department of Emergency The Affiliated Huai’an No. 1 People’s Hospital of Nanjing Medical University Huai’an China; ^3^ Department of Cardiology The Affiliated Suzhou Hospital with Nanjing Medical University Suzhou Municipal Hospital Gusu School of Nanjing Medical University Suzhou China; ^4^ Jiangsu Province Hospital Nanjing China; ^5^ Department of Rehabilitation Medicine School of Acupuncture‐Moxibustion and Tuina and School of Health Preservation and Rehabilitation Nanjing University of Chinese Medicine Nanjing China; ^6^ Department of Cardiology Shengli Clinical Medical College of Fujian Medical University Fujian Provincial Hospital Fuzhou University Affiliated Provincial Hospital Fuzhou China; ^7^ Fujian Provincial Key Laboratory of Cardiovascular Disease Fujian Cardiovascular Institute Fujian Clinical Medical Research Center for Cardiovascular Diseases Fuzhou China

**Keywords:** cold‐inducible RNA‐binding protein, KEAP1‐NRF2 pathway, oxidative stress, renal injury

## Abstract

Angiotensin II (Ang II) plays crucial roles in renal dysfunction, fibrotic changes, apoptosis, and oxidative stress. CIRBP, a cold‐inducible RNA‐binding protein, functions to maintain cellular homeostasis. However, its role in renal damage elicited by Ang II remains unclear. Here, we investigated the mechanisms through which *Cirbp* overexpression alleviates renal damage caused by Ang II, and *Cirbp* deletion induces basal renal injury. Transcriptomic analysis identified the involvement of *Cirbp* in hypertension‐related renal injury. Across the animal and cell models, *Cirbp* overexpression attenuated Ang II‐induced renal dysfunction, fibrosis, apoptosis, and inflammation, whereas *Cirbp* deficiency aggravated basal injury and increased renal susceptibility to injury. CIRBP activated the KEAP1‐NRF2 antioxidant pathway to mitigate redox stress. This renoprotective effect was reversed following the application of the NRF2 inhibitor ML385, but was resumed by the NRF2 agonist RA839. Together, CIRBP modulates redox imbalance during Ang II‐mediated renal damage through KEAP1‐NRF2 signaling.

## Highlights

- *Cirbp* protects against Ang II‐induced renal injury, whereas *Cirbp* deficiency increases susceptibility to renal damage.
- CIRBP attenuates redox stress through the activation of KEAP1‐NRF2 signaling, thereby reducing renal fibrosis, apoptosis, and inflammation.
- Targeting the CIRBP/KEAP1‐NRF2 axis may be explored as a potential therapeutic strategy for hypertensive renal injury.

## Graphical abstract

*Cirbp* is a key regulatory factor in hypertensive renal injury. The CIRBP‐KEAP1‐NRF2 signaling pathway represents a therapeutic target for Ang II‐induced renal injury and redox imbalance.

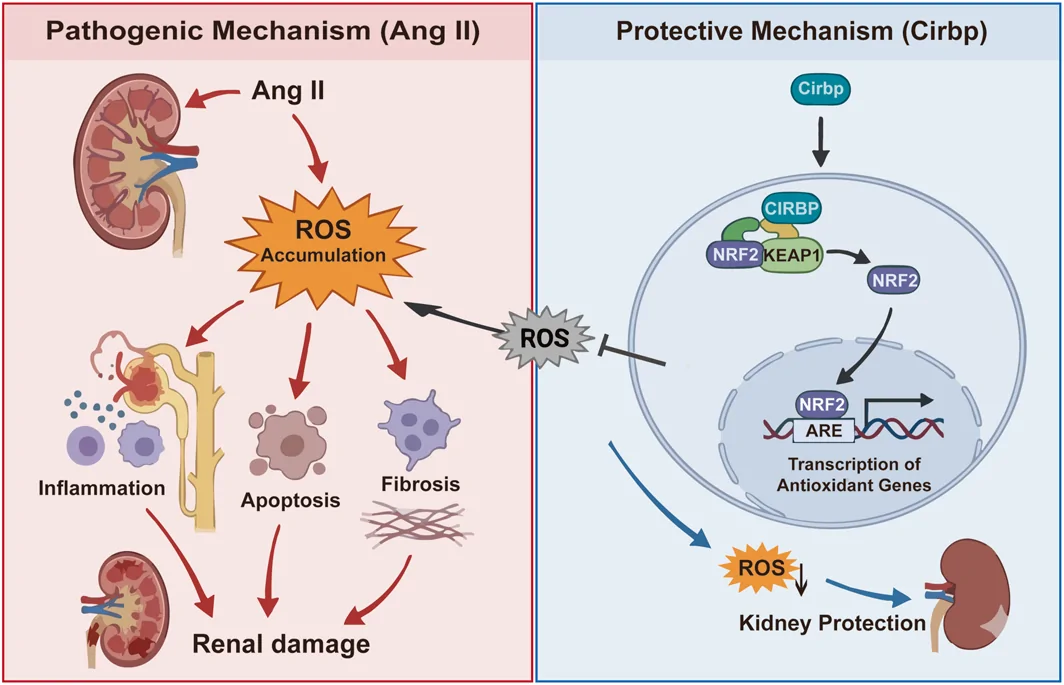

## 1 Introduction

Hypertensive renal injury contributes greatly to the worldwide burden of chronic kidney disease and end‐stage renal failure [1]. Its pathogenesis involves the renin‐angiotensin axis, within which angiotensin II (Ang II) exerts deleterious effects on renal function and structure. Ang II elevates arterial pressure by inducing vasoconstriction, disrupts water‐salt balance via the renal tubules, and stimulates aldosterone release [2, 3]. Ang II can also directly damage the kidney through imposing persistent oxidative injury [4]. Then, the sustained redox imbalance promotes vascular dysfunction and drives fibrotic and inflammatory cascades within the renal parenchyma [5]. Despite these findings, the endogenous counter‐regulatory mechanisms remain to be further explored and therapeutic strategies to be invented to reduce reactive oxygen species (ROS) accumulation.

Cold‐inducible RNA‐binding protein (*Cirbp*), which is a highly conserved stress‐responsive factor, regulates cellular adaptation to environmental assaults, including low temperatures, hypoxic exposure, ultraviolet radiation, and inflammation [6]. *Cirbp* guards cell survival through RNA stabilization, stress adaptation, and regulation of inflammatory signaling [7]. Recent studies have revealed that *Cirbp* participates in the cellular response to oxidative reactions in multiple types of tissues [8, 9, 10]. However, it remains unclear whether *Cirbp* affects Ang II‐induced antioxidant signaling pathways in the kidney.

The Kelch‐like ECH‐associated protein 1 (*Keap1*)‐nuclear factor erythroid 2‐related factor 2 (*Nrf2*) signaling is indispensable for adaptive cellular responses to oxidative stress [11]. Under a homeostatic condition, the NRF2 protein forms a complex with its endogenous inhibitory factor, KEAP1, which promotes rapid NRF2 turnover through the proteasome. Under oxidative conditions, a conformational change in KEAP1 releases NRF2 and facilitates NRF2 trafficking to the nucleus, where it upregulates transcription of antioxidant response element (ARE)‐dependent genes, thereby exerting cytoprotective effects [12]. Nrf2 protects from acute injury resulting from inflammation and oxidative stress [13], diabetic nephropathy [14], and hypertensive cardiomyopathy [15].

In the present work, we defined how *Cirbp* interacts with *Keap1*/*Nrf2* during Ang II‐induced renal injury and provided new mechanistic insights and potential therapeutic targets.

## 2 Methods

### 2.1 Animals and Modeling

CRISPR/Cas9 technology was used to modify the *Cirbp* gene. Guided by the structure of the *Cirbp* locus, the exon 7 of the *Cirbp*‐202 transcript (ENSMUST00000105365.9) was selected as the deletion target. This targeted sequence includes the initiation codon ATG, and its deletion disrupted protein function. *Cirbp* overexpression was achieved using AAV9 (1 × 10^12^ vg/mL; Genechem, Shanghai, China), and the corresponding control virus was injected via the tail vein.

*Cirbp*‐deficient mice (C57BL/6N) and *Cirbp*‐overexpressing mice (C57BL/6N), together with wild‐type (C57BL/6N) littermates, were maintained at the Experimental Animal Center of Nanjing Medical University, with unrestricted access to chow (AIN‐93M, Research Diet, < 0.3% NaCl) and water. Then, the animals were randomized to either a saline‐treated group or an Ang II (1.44 mg/kg/day; Sigma) group, and Ang II was administered via an Alzet osmotic minipump (Model 2004, California, USA). The pump was implanted subcutaneously in the dorsal region and retained for 4 weeks. The study comprised six groups: saline alone, Ang II alone, *Cirbp*‐OE, *Cirbp*‐OE + Ang II, *Cirbp*‐KO, and *Cirbp*‐KO + Ang II.

We performed all animal experiments following the National Institutes of Health Guide for the Care and Use of Laboratory Animals. The study protocol was approved by the Ethics Committee at Nanjing Medical University (Nanjing, China). Isoflurane inhalation was used for anesthesia, with 3% for induction and 1%–2% for maintenance. Euthanasia was performed by administering sodium pentobarbital intraperitoneally at a dose of 0.2 g/kg (Sigma, USA).

### 2.2 Histopathological Examination

Renal tissues were sectioned and dehydrated sequentially in ethanol solutions at gradient concentrations (75%, 80%, 90%, and 90%), followed by two additional rounds of dehydration in 100% ethanol for 1.5 h each. After two xylene‐clearing steps, the tissues were paraffin embedded and sections were prepared using a microtome (RM2016; Leica, Shanghai, China).

Masson trichrome staining was applied to the specimens according to the manufacturer’s instructions with a commercial kit (ServiceBio, Wuhan, China). Sections were first dried at 65°C for 2 h to enhance adhesion to the slides, followed by paraffin removal and graded rehydration. Following staining, slides were mounted with neutral gum. Micrographs were acquired using an optical microscope (Nikon, Japan).

Hematoxylin‐eosin (H&E) staining was used to visualize renal injury. Paraffin‐embedded sections were warmed for 2 h at 65°C, then deparaffinized and rehydrated in xylene and graded ethanol, followed by staining with hematoxylin solution for 3 min and rinsing in tap water. The sections were differentiated for 3–5 s in differentiation solution, and then underwent bluing for 3–6 s. The sections next underwent a 1‐min dehydration in 95% ethanol and were stained using eosin solution for 15 s. The tissues were subsequently dehydrated through 1‐min absolute ethanol I, absolute ethanol III, absolute ethanol I, n‐butanol II, and xylene I, followed by mounting in neutral gum. Images were collected using a Nikon microscope.

### 2.3 Immunofluorescence Analysis

Kidney transverse sections were embedded within frozen medium before storage at −80°C. Sections were equilibrated and washed, then underwent fixation with 4% paraformaldehyde and permeabilized in 0.2% Triton X‐100, followed by blocking nonspecific antigenic sites in 10% goat serum (ZLI‐9056; Origene Co., Beijing, China). The sections were incubated on a rocking chair for 12–16 h at 4°C with antibodies against CIRBP (rabbit, 1:200; sc‐293325; Santa Cruz), KEAP1 (mouse, 1:200; 60027‐1‐Ig; Proteintech), NRF2 (mouse, 1:200; YP‐mAb‐01916; UpingBio), or iNOS (rabbit, 1:200; 22226; Proteintech). After three PBS washes, antibodies were combined for 2 h with Cy3 or 488‐labeled IgG (1:100; GB21303, GB25301; Servicebio). DAPI (1:1000; MBD‐0015; Sigma‐Aldrich) was used for nuclear counterstaining. Fluorescence intensity was measured using ImageJ software from images acquired using an immunofluorescence microscope (Zeiss, Germany).

### 2.4 Renal Blood Flow Assessment by Laser Speckle Contrast Imaging

Renal blood flow was quantified using a MoorFLPI‐2 system (Moor Instruments Ltd., Devon, UK). Mice were anesthetized, and physiological temperature was maintained under constant‐temperature conditions. The kidneys were exposed and covered with gauze moistened with sterile saline maintained at 37°C. Indoor ambient lighting was standardized, and the zoom and focus of the imaging laser were optimized for high‐resolution images.

### 2.5 Cell Transfection and Treatment

Normal rat kidney cells (NRK‐52E) were revived and grown in 10% fetal bovine serum (Oricell, Shanghai, China) with DMEM (Gibco, USA) at 37°C under 5% CO2. For experiment, small interfering RNA (siRNA) targeting *Cirbp* (si‐*Cirbp*; 75 nM; RiboBio, GGTCCTACAGAGACAGCTA), *Keap1* (si‐*Keap1*, CCAATTCATGGCCCACAAA), an overexpression plasmid (*Cirbp*‐OE; 1 μg/mL; Jikai Gene, Shanghai, China), together with their corresponding controls, were transfected into the cells for 6–8 h using Lipofectamine 3000 (1:1000; L3000015; Invitrogen, MA, USA) and Opti‐MEM (1:100; Oricell, Shanghai, China). The cells were subsequently exposed to Ang II (1 μM; Bachem, CA, USA) for 48 h. For functional rescue experiments, ML385 (5 μM; HY‐100523, MCE, NJ, USA) or RA839 (5 μM; HY‐110275, MCE, NJ, USA) was added at the same time.

### 2.6 Western Blotting

Tissues and cells underwent lysis using a total protein extraction kit (KeyGen, China). Concentration of protein was quantified with a BCA assay (Thermo Fisher). The protein solution was mixed with loading buffer (FUDE Biological Technology, Zhejiang, China) and thermal denaturation for 10 min at 100°C. Following separation of 20 μg of protein sample on 10% SDS‐PAGE gels, the separated proteins were transferred to PVDF membranes. Similar to fluorescent staining, proteins were blocked and incubated with primary antibodies against CIRBP, COL III (1:1000; 22734, Proteintech), COL Ia1 (1:1000; BS1530, Bioworld), α‐SMA (1:1000; 14395, Proteintech), caspase‐3 (1:1000; 19677, Proteintech), cleaved caspase‐3 (1:1000; 9664T, CST), BCL‐2 (1:1000; AF6139, Affbiotech), BAX (1:1000; T40051s, Abmart), iNOS (1:1000; 22226, Proteintech), IL‐6 (1:1000; TD6087F, Abmart), HO‐1 (1:1000; TN24541F, Abmart), and GAPDH (1:10000; 60004‐1‐Ig, Proteintech). Next, membranes were exposed to HRP‐conjugated antibodies (1:10000; 115‐035‐003, 111‐035‐003, Jackson ImmunoResearch) for 2 h. Protein bands were detected on an iBright CL1000 platform (Invitrogen, MA, USA) with an enhanced chemiluminescence (ECL) kit (FUDE Biological Technology, Zhejiang, China).

### 2.7 Quantitative Real‐Time PCR (qRT‐PCR)

RNAiso (108‐95‐2; Takara) combined with chloroform was used to extract total RNA from kidneys and NRK. RNA concentration was measured using a NanoDrop instrument. Subsequently, 400 ng of mRNA was converted to complementary DNA (cDNA) in a 10‐μL reaction system with Hifair III Reverse Transcriptase (13762ES60; Yeasen, Shanghai, China) and a Bio‐Rad reverse transcription instrument. Quantitative PCR was subsequently performed with gene‐specific primers (Supporting Information S1: Table S1) and Hieff qPCR SYBR Green Mix (11201ES08; Yeasen) on a Real‐Time PCR System (ABI 7900HT, Applied Biosystems, USA). mRNA expression was normalized to GAPDH and quantified using the 2^−ΔΔCt^ method.

### 2.8 TUNEL Analysis

Apoptotic cells were detected with the YSFluor 488 TUNEL Kit (40307ES60; Yeasen). Sections or cultured cells were fixed and subjected to 10‐min permeabilization using 20 μg/mL proteinase K. The sample preparations were then reacted at 37°C for 1 h with the labeling compound, which consisted of equilibration buffer, YSFluor 488‐12‐dUTP, the enzyme of recombinant TdT, and double distilled water. Finally, all nuclei were counterstained with DAPI, after which TUNEL‐positive nuclei were quantified in ImageJ.

### 2.9 Measurement of Intracellular ROS

2′,7′‐Dichlorofluorescein diacetate (DCFH‐DA) and dihydroethidium (DHE) probes were used to measure ROS levels in cells and kidneys. Freshly prepared kidney sections were incubated with DCFH‐DA (10 μM; A147250310; Beyotime) and DHE (10 μM; S0064S; Beyotime) probes for 20 min at room temperature under light‐protected conditions. DAPI staining was then performed for 15 min before fluorescence imaging. For cell‐based assays, the probes were prepared in DMEM and applied together with Hoechst 33342 (1:100; C1028; Beyotime) in an incubator. After the cells underwent two rounds of washing with PBS for 5 min each, fluorescence signals were measured.

### 2.10 Co‐Immunoprecipitation (Co‐IP)

Co‐IP was conducted using an IP/CoIP Kit (Magnetic Beads) (KM0134; Dia‐An Biotechnology, Wuhan, China). Cells were lysed, centrifuged, and the supernatant was collected. In parallel, protein A/G magnetic beads were preincubated for 30 min with Flag and IgG antibodies (20543‐1‐AP and 30000‐0‐AP; Proteintech). After centrifugation, the cell lysates were mixed with the processed magnetic beads and incubated for a further 30 min. After the combination step was completed, antigen‐antibody complex was eluted with loading buffer by boiling. After Western blotting, bands were visualized using an iBright CL1000 system.

### 2.11 Cycloheximide (CHX) Chase Assay

KEAP1 protein stability was measured by the CHX chase assay. Following plasmid transfection, cells were stimulated with Ang II, after which the culture medium was exchanged for fresh DMEM supplemented with CHX (100 μg/mL; HY‐12320, MCE). The time nodes were set as 0, 1, 2, 4, 6, and 8 h, and temporal changes in KEAP1 protein were detected via Western blotting.

### 2.12 Cytoplasmic and Nuclear Protein Extraction

NRF2 accumulation in cytoplasmic and nuclear cells was evaluated with a commercial fractionation kit (P0027; Beyotime). Briefly, cells underwent two rounds of washing in ice‐cold PBS before resuspension in cytoplasmic extraction reagent containing PMSF. After a 15‐min incubation on ice, the samples were vortexed vigorously and centrifuged. The resulting supernatant was immediately retained as the cytoplasmic fraction, and the pellet was treated with nuclear extraction reagent containing PMSF and then incubated for 30 min on ice with intermittent vortex. Nuclear fraction refers to the supernatant after centrifugation. Nuclear marker anti‐Histone 3 (1:1000, 68345‐1‐Ig, Proteintech), and the cytoplasmic marker anti‐GAPDH were used as references in Western blotting.

### 2.13 RNA‐Sequencing and Data Analysis

The published RNA‐seq and single‐cell RNA‐seq data presented in the current article are publicly downloaded from the GEO database (https://ncbiinsights.ncbi.nlm.nih.gov/tag/geo/). Transcriptomic profiling was performed using the limma package in R v4.5.2. Re‐analysis of the mouse kidney single‐cell atlas followed the original quality control and clustering framework. Cells with 200–3000 detected genes and < 50% mitochondrial reads were retained (Supporting Information S1: Figure S1A). Data were log‐normalized to 1e4 molecules per cell, regressed for total UMI counts using the Seurat package, and batch‐corrected using the removeBatchEffect function of edgeR. A density‐based spatial clustering algorithm identified 16 robust cell populations. Cluster‐specific markers were defined by a two‐stage Seurat differential expression test to assign canonical renal cell identities, and *Cirbp* expression was visualized across the annotated renal cell populations.

### 2.14 Statistics and Reproducibility

Data are presented as the mean ± SEM. An unpaired *t* test was performed for the between‐group comparison. For multi‐group comparisons, a one‐ or two‐way ANOVA was performed according to the number of variances, with Tukey’s multiple‐comparison test then applied. A *p* < 0.05 was considered statistically significant. Statistical analysis and graph plotting were conducted using GraphPad Prism v10.1.2 software (Boston, MA, USA).

## 3 Results

### 3.1 Decrease of Cirbp Expression in Ang II‐Induced Renal Injury

RNA‐seq datasets, all including relatively large kidney sample sets from humans (GSE37463), mice (GSE207102), and rats (GSE311806), were selected (Figure 1A). Compared with control samples, 112 genes were consistently upregulated or downregulated in hypertension‐, Ang II‐, and high salt‐induced samples (*p* < 0.05). Gene Ontology (GO) enrichment (*p* < 0.05) indicated that these 112 genes were predominantly involved in protein binding, oxidoreductase activity, isomerase activity, and nicotinamide adenine dinucleotide phosphate (NADPH) binding (Figure 1B). According to false discovery rate (FDR) < 0.05, 19 differentially expressed genes (DEGs) were screened out, as shown in the heatmap (Figure 1C and Supporting Information S1: Table S2). Across all three species, *Cirbp* expression was reduced (Figure 1D) and was associated with oxidoreductase activity, a molecular function implicated in renal injury. Single‐cell transcriptomic analysis of normal mouse kidneys [16] identified 16 cell clusters, with proximal tubules accounting for 60.54% (Figure 1E and Supporting Information S1: Figure S1B). According to *Cirbp* staining results (Figure 1F), the average *Cirbp* expression level in proximal tubules was 0.152 and the proportion of *Cirbp*‐positive cells was 9.64% (Supporting Information S1: Figure S1C). Total *Cirbp* expression was the highest in proximal tubules and this type of cells was a principal target of Ang II (Figure 1G and Supporting Information S1: Figure S1D). Therefore, the rat kidney‐derived NRK‐52E proximal tubular epithelial cell line was selected for subsequent analysis.

**FIGURE 1:**
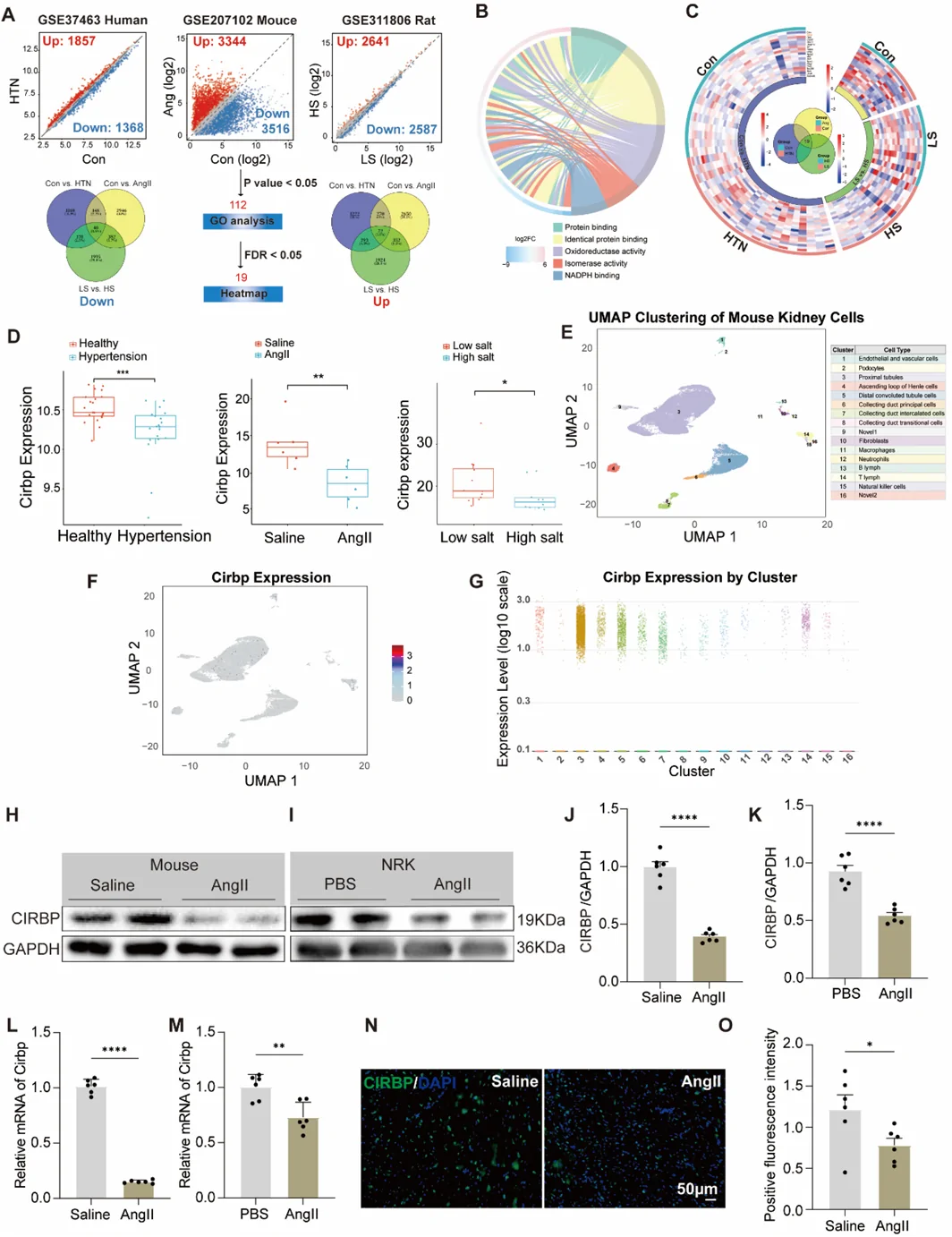
Decrease of *Cirbp* expression during Ang II‐mediated renal injury. (A) Schematic illustration depicting the renal RNA‐seq analysis strategy. GSE37463 includes patients with hypertensive nephropathy (*n* = 20) and healthy kidneys (*n* = 21). GSE207102 includes Ang II‐induced mice (*n* = 6) and controls (*n* = 6). GSE311806 includes rats receiving low‐ (*n* = 12) and high‐salt water (*n* = 12). Con, control; HTN, hypertension; HS, high‐salt drinking water; LS, low‐salt water. (B) GO analysis of 112 genes shared by the three datasets (*p* < 0.05). (C) Circular heatmap of 19 genes differentially expressed in all three datasets (FDR < 0.05). (D) Transcriptional expression of *Cirbp* in the three RNA‐seq datasets. (E) Single‐cell transcriptomic analysis of mouse kidney samples in GSE107585. Unsupervised analysis identified 16 cell populations displayed on a UMAP plot. (F) *Cirbp* staining in (E). (G) Cluster‐based distribution of *Cirbp* expression. (H, I) Western blotting assessment of CIRBP protein abundance in kidneys from WT mice and in NRK‐52E receiving saline or Ang II (*n* = 6). (J, K) Quantification of protein levels in (H, I). (L) qRT‐PCR quantification of *Cirbp* transcripts in kidney obtained from WT mice given saline or exposed to Ang II (*n* = 6). (M) qRT‐PCR quantification of *Cirbp* transcripts in NRK‐52E cells after PBS or Ang II exposure (*n* = 6). (N, O) Representative immunofluorescence micrographs showing CIRBP staining in kidneys from WT mice following Ang II administration (*n* = 6). *p < 0.05, **p < 0.01, ***p < 0.001, ****p < 0.0001.

To validate the results from RNA‐seq, *Cirbp* expression was evaluated on protein and mRNA levels in kidneys from C57BL/6N mice receiving Ang II and in NRK cells following Ang II treatment. In comparison with controls, CIRBP protein levels were lower in the kidneys with Ang II stimulation (Figure 1H,J), as well as in Ang II‐treated NRK cultures (Figure 1I,K). Similarly, *Cirbp* mRNA expression decreased significantly in both kidneys (Figure 1L) and cells (Figure 1M) after Ang II treatment. Immunofluorescence staining further showed a reduction in CIRBP expression in kidneys from Ang II‐infused animals relative to controls (Figure 1N,O).

### 3.2 Effects of *Cirbp* Knockout and Overexpression on Ang II‐Mediated Kidney Damage and Renal Dysfunction

The protective actions of *Cirbp* were further investigated using *Cirbp*‐OE and *Cirbp*‐KO models. We confirmed *Cirbp* expression in kidneys from Cirbp‐KO mice; the findings showed that CIRBP protein abundance was significantly reduced in *Cirbp*‐KO mice relative to wild‐type (WT) mice (Supporting Information S1: Figure S2A,B). At 4 weeks after viral injection, CIRBP protein expression increased in kidneys from AAV9‐*Cirbp*‐injected mice relative to control mice (Supporting Information S1: Figure S2C,D). These findings indicated that the *Cirbp*‐KO and *Cirbp*‐OE mice were suitable for subsequent experiments. In the formal experiment, after 1 week of acclimation, *Cirbp*‐OE mice and the corresponding control mice received tail vein injection of AAV9. After a sufficient period for AAV9‐mediated expression, Ang II was infused subcutaneously for 4 weeks via an implanted minipump (Figure 2A). At the experimental endpoint, serum and kidney samples were collected. The concentrations of serum creatinine (CREA) and blood urea nitrogen (BUN) were elevated in the Ang II group, whereas *Cirbp* overexpression attenuated these changes. Compared with WT mice, *Cirbp*‐KO mice exhibited higher baseline CREA and BUN levels, without a further increase after Ang II infusion (Figure 2B,C). H&E staining revealed that Ang II caused glomerular edema and interstitial disorganization. *Cirbp* overexpression attenuated Ang II‐induced pathological damage, whereas *Cirbp* deficiency aggravated baseline injury (Figure 2D,E). Laser speckle contrast imaging showed that renal blood flow was reduced after Ang II infusion. *Cirbp* overexpression reversed Ang II‐induced reduction in renal blood flow, whereas *Cirbp* deficiency reduced basal renal perfusion (Figure 2F,G). In *Cirbp*‐OE mice, the Ang II‐induced enrichment of the renal dysfunction marker neutrophil gelatinase‐associated lipocalin (*Ngal*) was significantly attenuated (Figure 2H), whereas *Cirbp* knockout increased *Ngal* expression (Figure 2I). Blood pressure was measured every week post‐Ang II administration using the tail‐cuff technique (Figure 2J). At the last week, *Cirbp* overexpression reduced Ang II‐induced elevated systolic blood pressure, but not diastolic and mean blood pressure. The deletion of *Cirbp* led to higher blood pressure with or without Ang II infusion.

**FIGURE 2:**
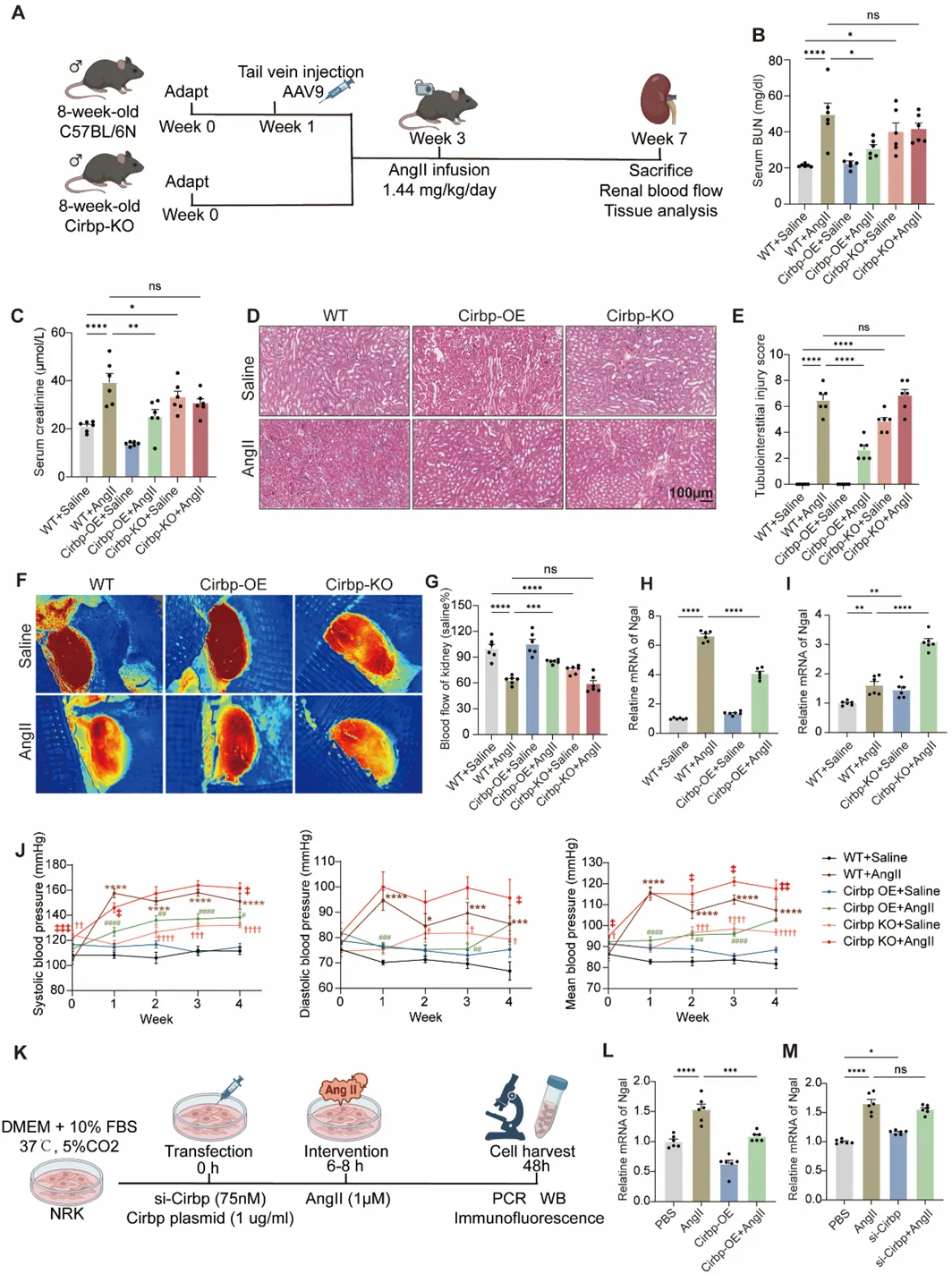
Differential consequences of *Cirbp* knockout and overexpression during Ang II‐mediated renal injury and dysfunction. (A) Flowchart of the mice experimental procedures. (B) ELISA of BUN levels in serum from WT, *Cirbp*‐OE, and *Cirbp*‐KO mice given saline or treated with Ang II (*n* = 6). BUN, blood urea nitrogen. (C) ELISA measuring serum creatinine levels in WT, *Cirbp*‐OE, and *Cirbp*‐KO mice receiving Ang II (*n* = 6). (D, E) H&E‐stained kidneys from WT, Cirbp‐OE, and Cirbp‐KO mice receiving saline or Ang II and measurement of tubulointerstitial injury (*n* = 6). (F, G) Images from laser speckle blood flow analysis of kidneys in living mice. Darker red indicates greater blood flow (*n* = 6). (H, I) qRT‐PCR quantification of *Ngal* transcripts in kidneys from WT, *Cirbp*‐OE, and *Cirbp*‐KO mice (*n* = 6). (J) Blood pressure measurements in WT, *Cirbp*‐OE, and *Cirbp*‐KO mice (*n* = 6). *WT + Ang II versus WT + Saline; ^†^Cirbp‐KO + Saline versus WT + Saline; ^#^Cirbp‐OE + Ang II versus WT + Ang II; ^‡^Cirbp‐KO + Ang II versus WT + Ang II. (K) Flowchart of the cell experimental procedures. (L, M) qRT‐PCR quantification of *Ngal* transcripts in NRK cultures transfected with negative control, *Cirbp*‐OE, or si‐*Cirbp* after PBS or Ang II (*n* = 6).

We next examined whether *Cirbp* overexpression by plasmid (*Cirbp*‐OE) or *Cirbp* knockdown by siRNA (si‐*Cirbp*) relieved renal cell injury in vitro. After 6–8 h of transfection, *Cirbp* mRNA and protein levels were confirmed in NRK cells. siRNA transfection reduced *Cirbp* mRNA expression in NRK cells (Supporting Information S1: Figure S3A), whereas plasmid transfection increased *Cirbp* mRNA expression (Supporting Information S1: Figure S3B). Similar changes were observed at the protein level. *Cirbp* expression increased after plasmid transfection (Supporting Information S1: Figure S3C,D) and decreased after siRNA transfection (Supporting Information S1: Figure S3E,F). Following 48 h of Ang II exposure (Figure 2K), the Ang II‐induced increase in *Ngal* transcripts in NRK cells was reversed by *Cirbp* overexpression (Figure 2L), whereas *Cirbp* knockdown further increased *Ngal* mRNA expression (Figure 2M). Together, *Cirbp* overexpression ameliorated Ang II‐mediated kidney injury and dysfunction, whereas *Cirbp* knockdown aggravated baseline renal injury.

### 3.3 Impact of *Cirbp* on Ang II‐Induced Renal Fibrotic Changes

Masson staining showed that after continuous infusion of Ang II, renal fibrosis was more serious in *Cirbp*‐KO mice relative to wild‐type (WT) mice. *Cirbp* deficiency also induced renal fibrosis under physiological conditions. In contrast, *Cirbp* overexpression reduced the blue collagen‐positive area in Ang II‐treated kidneys (Figure 3A,B). In addition, Ang II‐associated elevations in the mRNA abundance of the fibrosis‐associated markers alpha‐smooth muscle actin (*α‐sma*) and collagen type I (*Col I*) were attenuated in *Cirbp*‐OE mice (Figure 3C), but aggravated in *Cirbp*‐KO mice (Figure 3D). In line with the transcript profiles, immunoblotting demonstrated that after Ang II infusion, the protein levels of collagen III (COL III), collagen I alpha 1 chain (COL Ia1), and α‐SMA were reduced in *Cirbp*‐OE mice (Figure 3E,F). Levels of the same fibrosis markers were also significantly greater in *Cirbp*‐KO mice than in WT mice (Figure 3G,H).

**FIGURE 3:**
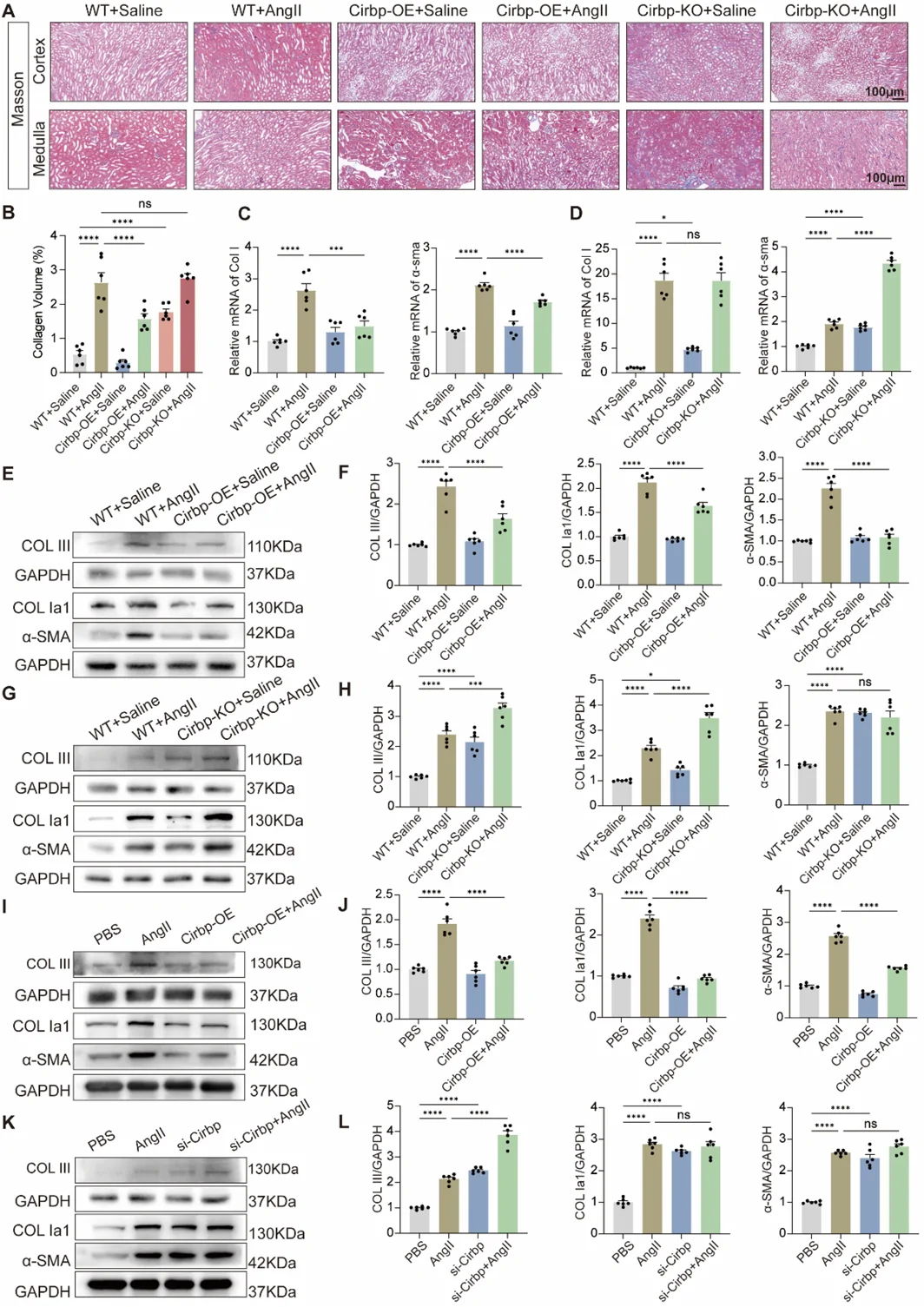
*Cirbp* modulates Ang II‐associated renal fibrosis. (A, B) Masson trichrome staining of cortical and medullary fibrosis in WT, Cirbp‐OE, and Cirbp‐KO mice (*n* = 6). (C, D) qRT‐PCR‐based quantification of *Col I* and *α‐sma* transcripts in kidneys from WT and *Cirbp*‐OE or *Cirbp*‐KO mice (*n* = 6). (E, F) Immunoblot analysis of COL III, COL Ia1, and α‐SMA protein levels in kidneys from WT and Cirbp‐OE kidneys given saline or Ang II treatment (*n* = 6). (G, H) Western blotting analysis of COL III, COL Ia1, and α‐SMA protein levels in kidneys from WT and Cirbp‐KO mice (*n* = 6). (I, J) Western blotting analysis of COL III, COL Ia1, and α‐SMA abundance in NRK cells with Cirbp‐OE after PBS or Ang II stimulation (*n* = 6). (K, L) Western blotting assessment of COL III, COL Ia1, and α‐SMA abundance in NRK cells with si‐Cirbp after PBS or Ang II stimulation (*n* = 6).

We next investigated whether *Cirbp* exerts similar antifibrotic effects in vitro under neurohumoral renal stress. At the transcript level (Supporting Information S1: Figure S4A) and at the protein level (Figure 3I,J), *Cirbp*‐OE NRK cells showed milder fibrosis following Ang II treatment. Compared to the control without Ang II stimulation, si‐*Cirbp* increased fibrosis‐related transcripts (Supporting Information S1: Figure S4B) and proteins (Figure 3K,L) associated with cellular fibrosis. These results indicate that *Cirbp* overexpression attenuated renal fibrosis during Ang II exposure, whereas *Cirbp* deficiency elicited fibrosis under physiological conditions.

### 3.4 Effects of *Cirbp* on Ang II‐Mediated Renal Cell Apoptosis

Ang II damages podocytes and promotes apoptosis both in culture and in vivo. Terminal deoxynucleotidyl TUNEL staining showed that renal apoptosis was significantly worsened following Ang II infusion but was reduced after *Cirbp* overexpression. Compared with WT mice, *Cirbp* deficiency resulted in basal renal apoptosis (Figure 4A,B). Similarly, the in vitro TUNEL staining results were consistent with those observed in the animal experiments, regardless of whether *Cirbp* was overexpressed (Figure 4C,D) or deficient (Figure 4E,F). As expected, after Ang II infusion, the apoptosis indicators cleaved caspase 3/caspase 3 together with B‐cell lymphoma 2 (Bcl‐2)/BCL‐2‐associated X protein (*Bax*) were reduced in *Cirbp*‐OE mice at the transcript level (Supporting Information S1: Figure S5A) or at the protein level (Figure 4G,H). These indicators did not further increase in *Cirbp*‐KO mice receiving Ang II treatment; however, *Cirbp* deficiency increased the expression of apoptosis markers compared with WT mice at the transcript level (Supporting Information S1: Figure S5B) and at the protein level (Figure 4I,J). In vitro, *Cirbp* exerted the same anti‐apoptotic effect. *Cirbp*‐OE attenuated Ang II‐induced apoptosis at the transcript level (Supporting Information S1: Figure S5C) and at the protein level (Figure 4K,L). *Cirbp* deficiency further increased apoptosis marker expression at the transcript level (Supporting Information S1: Figure S5D) and at the protein level (Figure 4M,N), irrespective of Ang II exposure. These findings support the view that overexpression of *Cirbp* within the kidney could provide an effective strategy for reversing renal apoptosis.

**FIGURE 4:**
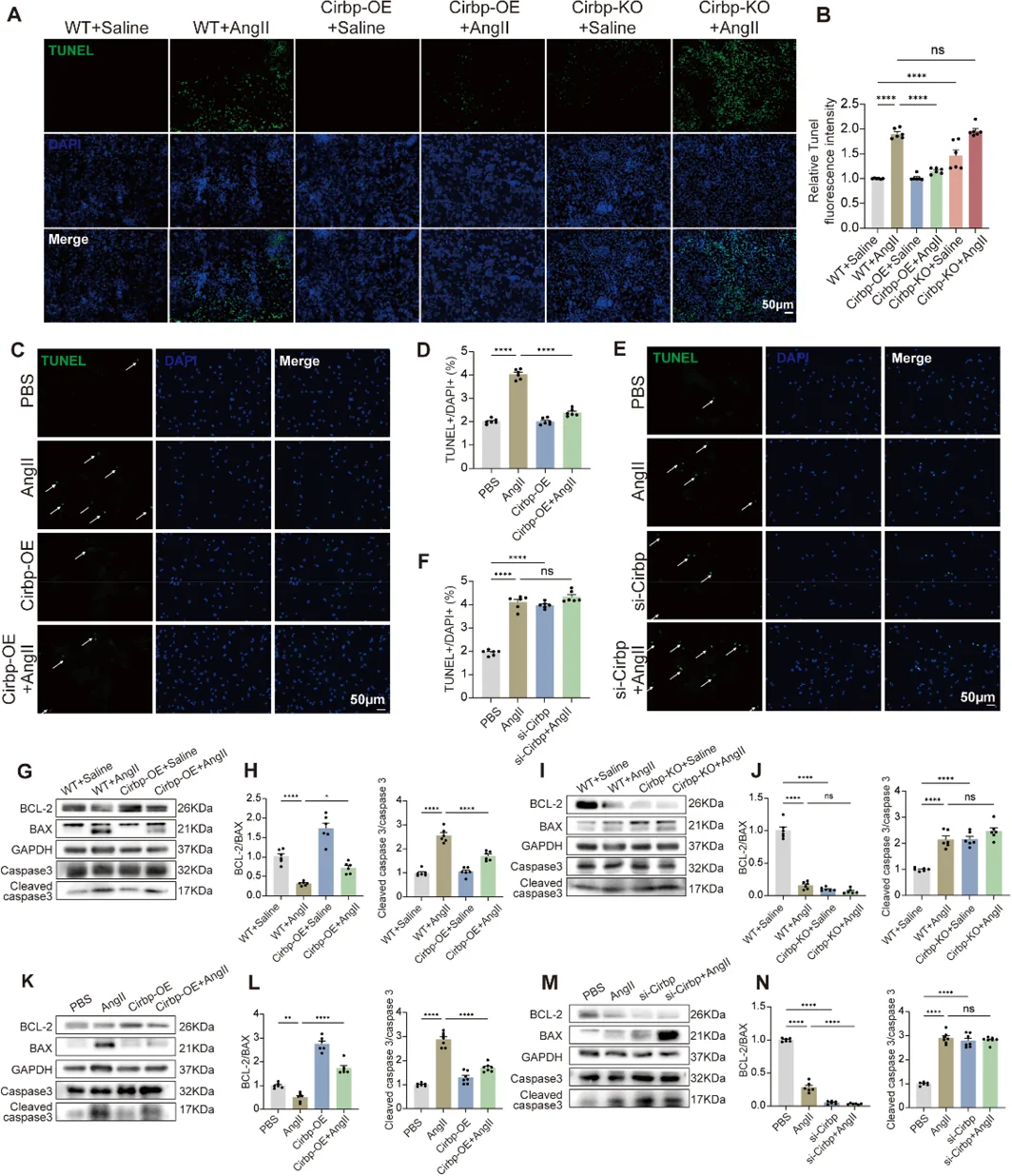
Effects of *Cirbp* on Ang II‐mediated renal apoptosis. (A, B) TUNEL staining showing apoptosis within kidneys from WT, *Cirbp*‐OE and *Cirbp*‐KO mice receiving Ang II treatment (*n* = 6). (C, D) TUNEL staining showing apoptosis in NRK cells with *Cirbp*‐OE after Ang II stimulation (*n* = 6). (E, F) TUNEL staining of NRK with si‐*Cirbp* after Ang II (*n* = 6). (G, H) Western blotting assessment of apoptosis‐related protein abundance in kidneys from WT and *Cirbp*‐OE mice kidney (*n* = 6). (I, J) Corresponding Western blotting analysis in WT and Cirbp‐KO kidneys (*n* = 6). (K, L) Western blotting assessment of apoptosis‐related protein expression in NRK cells with *Cirbp*‐OE after PBS or Ang II stimulation (*n* = 6). (M, N) Corresponding Western blotting analysis in si‐Cirbp NRK cells (*n* = 6).

### 3.5 Impact of *Cirbp* on Ang II‐Driven Renal Inflammatory Responses

Ang II is also a proinflammatory stimulus and acts by directly driving inflammatory responses in the kidney through several signaling routes. In Ang II‐infused kidneys, the expression of the inflammatory markers inducible nitric oxide synthase (inos), tumor necrosis factor‐alpha (Tnf‐α), and interleukin‐6 (Il‐6) increased at both the transcript (Figure 5A) and protein (Figure 5B,C) levels, and both responses were attenuated after *Cirbp* overexpression. In contrast, renal inflammatory indices increased in *Cirbp*‐KO mice at the transcript level (Figure 5D) and at the protein level (Figure 5E,F), regardless of whether Ang II was administered. iNOS expression in the kidney has been reported to be positively correlated with the degree of glomerular injury [17].

**FIGURE 5:**
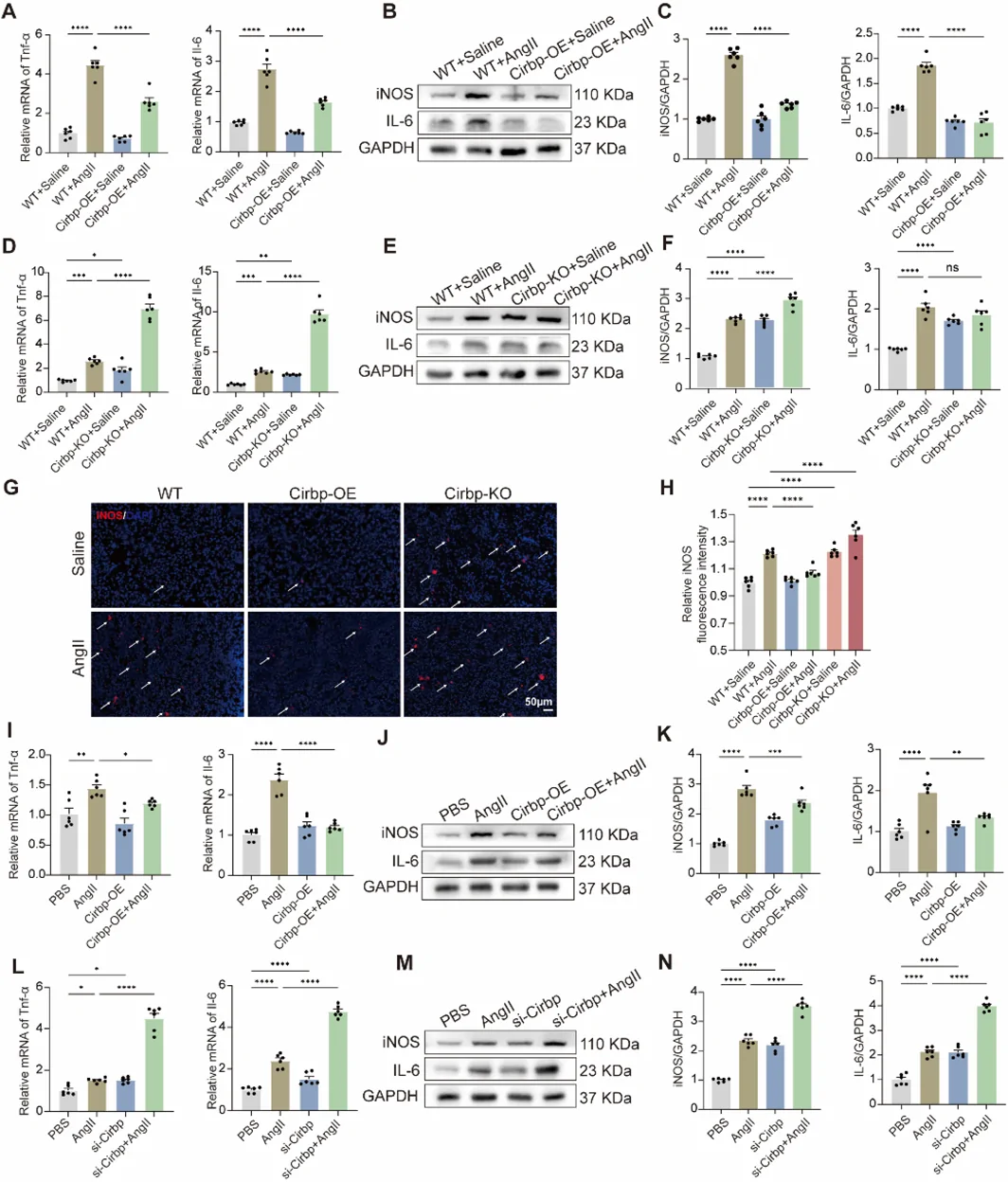
Cirbp suppresses Ang II‐associated renal inflammation. (A) qRT‐PCR‐based assessment of *Tnf‐α* and *Il‐6* mRNA in kidneys from WT and *Cirbp*‐OE mice given saline or Ang II (*n* = 6). (B, C) Western blotting assessment of iNOS and IL‐6 protein abundance in kidney tissue from WT and Cirbp‐OE mice (*n* = 6). (D) qRT‐PCR‐based quantification of *Tnf‐α* and *Il‐6* transcripts in kidneys from WT and *Cirbp*‐KO mice (*n* = 6). (E, F) Western blotting analysis of iNOS and IL‐6 protein abundance in kidney tissue from WT and *Cirbp*‐KO mice (*n* = 6). (G, H) Immunofluorescence micrographs showing iNOS signal in kidneys from WT, *Cirbp*‐OE and *Cirbp*‐KO mice (*n* = 6). (I) qRT‐PCR quantification of *Tnf‐α* and *Il‐6* transcripts in NRK cells with *Cirbp*‐OE after PBS or Ang II stimulation (*n* = 6). (J, K) Immunoblot assessment of iNOS and IL‐6 protein levels in NRK cells with *Cirbp*‐OE after PBS or Ang II stimulation (*n* = 6). (L) qRT‐PCR assessment of *Tnf‐α* and *Il‐6* transcripts in NRK cells with si‐*Cirbp* after PBS or Ang II (*n* = 6). (M, N) Western blotting analysis of iNOS and IL‐6 protein levels in NRK‐52E with si‐*Cirbp* after PBS or Ang II stimulation (*n* = 6).

Immunofluorescence staining demonstrated that the Ang II‐associated increase in iNOS was reduced within the kidneys of *Cirbp*‐OE mice, whereas *Cirbp* knockout further increased iNOS expression (Figure 5G,H). Consistent with the findings in animals, TNF‐α, IL‐6, and iNOS increased at the transcript level (Figure 5I) and at the protein levels (Figure 5J,K), following Ang II treatment of NRK cells. *Cirbp* knockdown further aggravated the inflammation at the transcript level (Figure 5L) and at the protein level (Figure 5M,N). Taken together, Cirbp was implicated in Ang II‐induced renal inflammation.

### 3.6 Alleviation on Renal Damage by Cirbp via Reducing Oxidative Stress

In the RNA‐seq data, 4294 upregulated genes and 3026 downregulated genes were detected (*p* < 0.05, |log_2_Foldchange| > 1). GO enrichment analysis of DEGs using the R package clusterProfiler showed their enrichment in pathways related to ROS and oxidative phosphorylation (Figure 6A). Complementary gene set enrichment analysis (GSEA) further provided evidence that Ang II promoted ROS generation (Figure 6B), and a heatmap was constructed to screen genes associated with ROS generation (Figure 6C). Together, these data implicate oxidative stress as a component of Ang II‐mediated renal injury.

**FIGURE 6:**
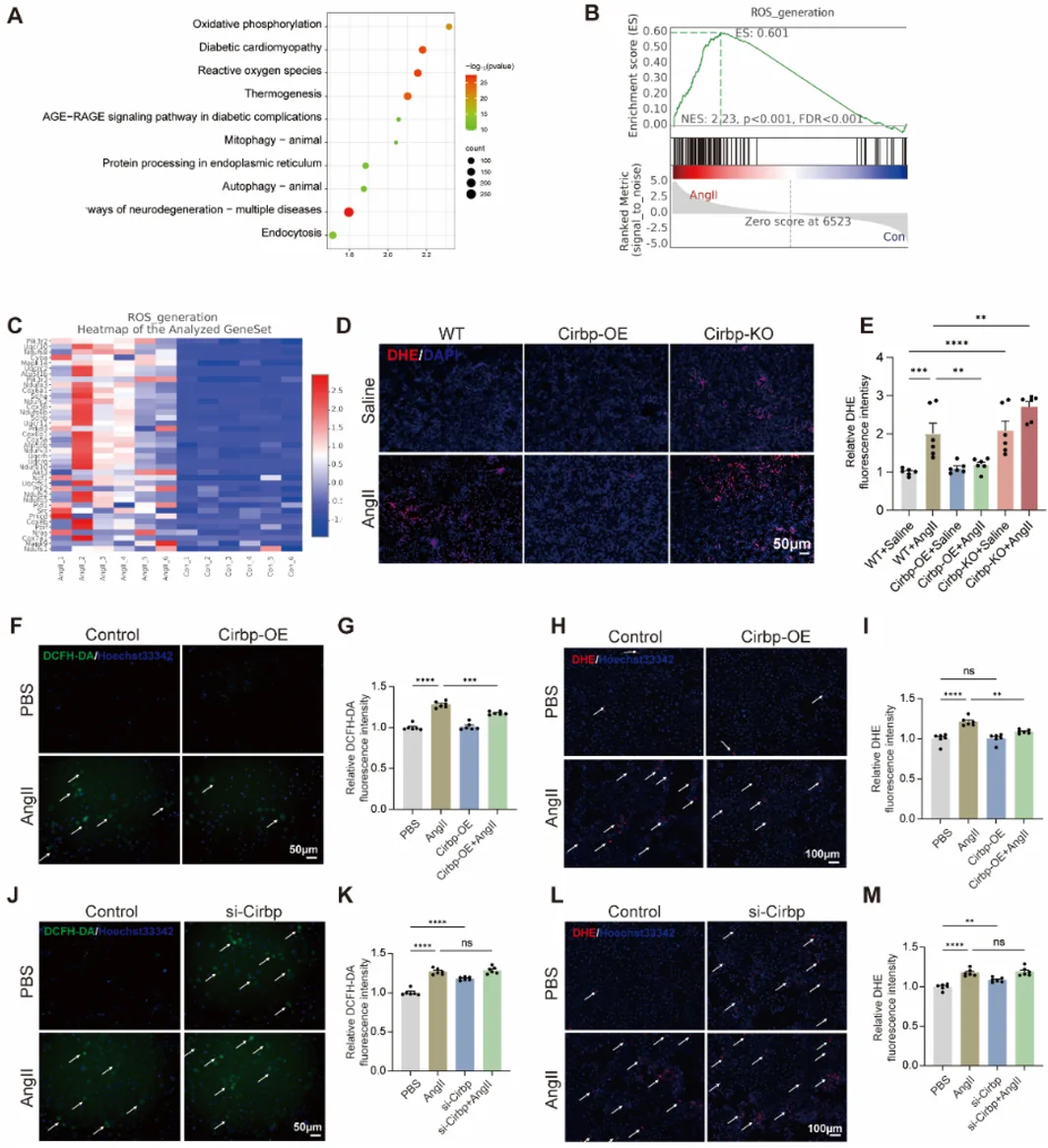
*Cirbp* regulates renal injury via oxidative stress. (A) KEGG enrichment of DEGs from the RNA‐seq (GSE207102) dataset derived from mice given saline or a continuous Ang II infusion (*n* = 6; |log2FC| > 1; *p* value < 0.05). (B) GSEA of ROS generation among mice given saline or infused with Ang II. (C) Heatmap of differentially expressed genes associated with ROS generation in mice administered saline or Ang II. (D, E) DHE labeling of ROS in kidneys from WT, *Cirbp*‐OE, and *Cirbp*‐KO mice (*n* = 6). (F, G) 2′,7′‐DCFH‐DA staining of ROS in NRK cells with *Cirbp*‐OE (*n* = 6). (H, I) DHE‐based detection of reactive oxygen species within NRK cultures with *Cirbp*‐OE (*n* = 6). (J, K) DCFH‐DA staining of ROS in NRK cells with *Cirbp*‐OE after PBS or Ang II stimulation (*n* = 6). (L, M) DHE staining of ROS in NRK cells with si‐*Cirbp* after PBS or Ang II stimulation (*n* = 6).

We further examined whether *Cirbp* affects Ang II‐induced oxidative stress. DHE labeling revealed that the rise in ROS elicited by Ang II was attenuated by *Cirbp*‐OE, but further enhanced by *Cirbp*‐KO (Figure 6D,E). Furthermore, both DCFH‐DA and DHE staining techniques were used to assess intracellular ROS levels in vitro. After Ang II stimulation, DCFH‐DA green fluorescence (Figure 6F,G) and DHE red fluorescence (Figure 6H,I), both reflecting the level of ROS, became shallower in *Cirbp*‐OE cells. *Cirbp* knockdown directly induced oxidative stress regardless of whether Ang II was present (Figure 6J–M). These results suggested that *Cirbp* ameliorated Ang II‐induced renal injury by alleviating oxidative stress.

### 3.7 Reduction in KEAP1 Protein Abundance by Cirbp Overexpression After Ang II Infusion in Renal Tissue

To define how *Cirbp* ameliorates Ang II‐mediated renal injury and redox imbalance and to identify the genes involved, we examined the DEGs described above and found that the mRNA expression of *Keap1*, an oxidative‐stress‐associated gene, was elevated following Ang II infusion in mice (Figure 7A). We then quantified the expression of *Keap1* and downstream antioxidant targets Heme Oxygenase‐1 (*Hmox1*) together with NADPH Quinone Oxidoreductase 1 (*Nqo1*). The qRT‐PCR showed that *Keap1* mRNA increased in Ang II infusion mice, while it maintained stability after changes in *Cirbp* (Supporting Information S1: Figure S6A,C). Ang II‐induced decreases in *Hmox1* and *Nqo1* mRNA were increased in the kidneys of *Cirbp*‐OE mice, while *Cirbp* knockout decreased their expression at the baseline level (Supporting Information S1: Figure S6B,D). This indicated that *Cirbp* did not regulate the expression of *Keap1* at the transcript level. An Ang II‐driven elevation in KEAP1 protein levels and a concomitant reduction in NRF2 and HO‐1 protein expression within renal tissue were reversed by *Cirbp*‐OE (Figure 7B,C), whereas *Cirbp*‐KO increased KEAP1 expression and lowered NRF2 and HO‐1 expression, contrary to the WT control (Figure 7D,E).

**FIGURE 7:**
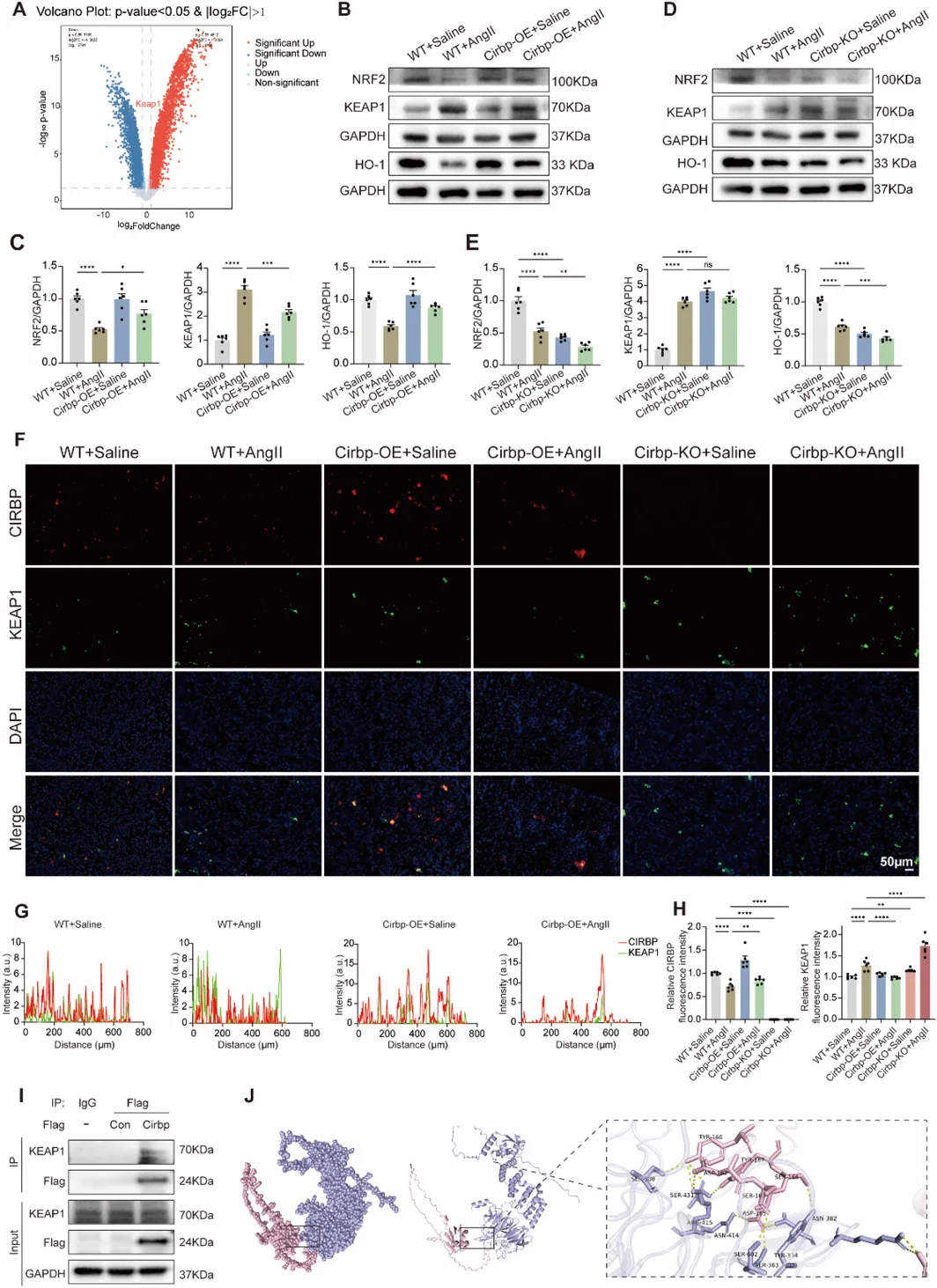
Overexpression of *Cirbp* decreases KEAP1 abundance in renal tissue after Ang II infusion. (A) Volcano plot depicting DEGs identified by RNA‐seq (GSE207102) in mice receiving saline or Ang II treatment (*n* = 6; |log2FC| > 1; *p* < 0.05). (B, C) Immunoblot assessment of KEAP1, NRF2 and HO‐1 protein abundance in kidneys from WT as well as *Cirbp*‐OE mice following Ang II infusion (*n* = 6). (D, E) Immunoblot assessment of KEAP1, NRF2 and HO‐1 protein levels in kidneys from WT and *Cirbp*‐KO mice administered Ang II (*n* = 6). (F) Representative immunofluorescence micrographs showing KEAP1 and CIRBP labeling in kidneys from WT, *Cirbp*‐OE, and *Cirbp*‐KO mice following Ang II administration (*n* = 6). (G) Fluorescence colocalization between KEAP1 and CIRBP in (F). (H) Quantification of fluorescence intensity in (F). (I) Co‐IP and Western blotting of CIRBP and KEAP1 in the transfected HEK293 cells. (J) Prediction of spatial configuration and peptide chain conformation of the CIRBP and KEAP1 binding by AlphaFold 3. Pink presents CIRBP (Uniprot ID: P60824) and blue represents KEAP1 (Uniprot ID: Q9Z2X8). Visualization of hydrogen bonds between KEAP1 and CIRBP and the corresponding amino acid residues by PyMOL software.

We next examined the relationship between CIRBP and KEAP1. Immunofluorescence staining showed partial colocalization in the kidney (Figure 7F,G), and fluorescence intensity analysis further confirmed the differential expression of KEAP1 in Ang II‐infused kidneys (Figure 7H). The Pearson’s correlation coefficient and M1 (fraction of CIRBP overlapping KEAP1) were decreased in *Cirbp*‐OE mice compared with the control group with or without Ang II infusion (*p* < 0.001), while the M2 coefficient (fraction of KEAP1 overlapping CIRBP) was the opposite. The Manders’ coefficients revealed that the extent to which CIRBP covers KEAP1 was weaker than that to which KEAP1 covers CIRBP (Supporting Information S1: Figure S6E). These quantitative data demonstrated the spatial proximity of CIRBP and KEAP1 in the kidney. AlphaFold 3 assessed the potential direct interaction between the two proteins (Supporting Information S1: Figure S6G). When *Cirbp* was transfected into HEK293 cells, Co‐IP showed that KEAP1 could directly bind to CIRBP (Figure 7I). Endogenous and reciprocal Co‐IP showed that CIRBP interacted with KEAP1 under physiological conditions (Supporting Information S1: Figure S6F). Molecular docking further displayed the spatial position between them. The hydrogen bonds and amino acid residues involved in forming the binding interface linking CIRBP and KEAP1 were mapped with the PyMOL software (Figure 7J). These results indicate that KEAP1 might act downstream of CIRBP in regulating renal oxidative stress.

### 3.8 Promotion of CIRBP on KEAP1 Degradation and NRF2 Functional Activation

To determine whether CIRBP regulated KEAP1 expression at the protein level, we performed a CHX chase assay. KEAP1 protein levels declined progressively over time in control cells treated with CHX, whereas *Cirbp* overexpression promoted KEAP1 degradation (Figure 8A,B) and shortened the half‐life (Supporting Information S1: Figure S7A). Given that KEAP1 is primarily degraded via the ubiquitin‐proteasome pathway, we next examined whether CIRBP affected KEAP1 ubiquitination. Co‐IP showed that KEAP1 ubiquitination was increased in *Cirbp*‐OE cells treated with the proteasome inhibitor MG132 relative to controls (Figure 8C). These observations suggest that CIRBP post‐transcriptionally regulates KEAP1 by promoting ubiquitination‐mediated proteasomal degradation.

**FIGURE 8:**
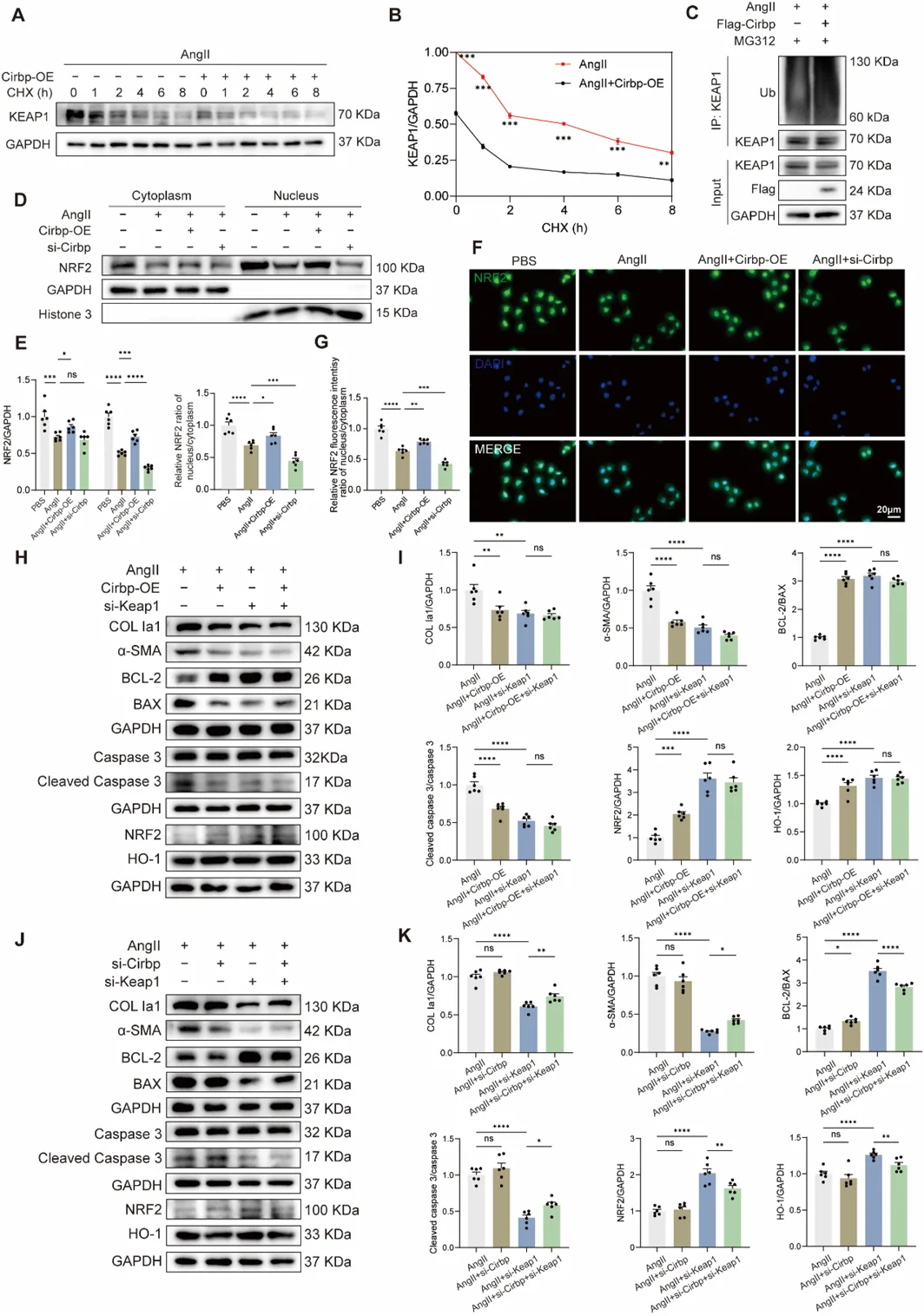
CIRBP promotes KEAP1 degradation and NRF2 functional activation. (A) Cycloheximide (CHX) chase assay showing KEAP1 protein stability in *Cirbp*‐OE NRK‐52E cells treated with CHX (0, 1, 2, 4, 6, 8 h) (*n* = 6). (B) Quantification of KEAP1 protein in (A). (C) Co‐immunoprecipitation assay detecting KEAP1 ubiquitination in *Cirbp*‐OE HEK293 cells with MG132 treatment. (D, E) Western blotting analysis of NRF2 protein in cytoplasmic and nuclear in NRK cells with si‐*Cirbp* or *Cirbp*‐OE after PBS or Ang II stimulation (*n* = 6). (F, G) Immunofluorescence staining of NRF2 showing cytoplasmic and nuclear localization in NRK cells with si‐*Cirbp* or *Cirbp*‐OE after PBS or Ang II (*n* = 6). (H, I) Western blotting analysis of proteins of fibrosis, apoptosis and oxidative stress markers in NRK‐52E with *Cirbp*‐OE or si‐*Keap1* after saline or Ang II infusion (*n* = 6). (J, K) Immunoblot assessment of COL Ia1, α‐SMA, BCL‐2, BAX, caspase 3, cleaved caspase 3, NRF2, and HO‐1 protein abundance in NRK‐52E with si‐*Cirbp* or si‐*Keap1* after PBS or Ang II stimulation (*n* = 6).

Since KEAP1 is a negative regulator of NRF2, we next investigated whether CIRBP‐mediated KEAP1 suppression affected NRF2 nuclear translocation. After cytoplasmic and nuclear protein extraction, Western blotting showed that NRF2 protein was enriched in the nuclear fraction in *Cirbp*‐OE cells and decreased in si‐*Cirbp* cells treated with Ang II (Figure 8D,E). Immunofluorescence staining of NRF2 revealed that *Cirbp* overexpression induced partial movement of NRF2 out of the cytoplasm and into the nucleus after Ang II infusion, whereas si‐*Cirbp* had the opposite effect (Figure 8F,G).

To confirm KEAP1 as a necessary mediator of the protective effects of CIRBP, *Keap1* was knocked down for a functional reversal experiment. The mRNA and protein levels verified the validity of si‐*Keap1* (Supporting Information S1: Figure S7B–D). Western blotting showed that si‐*Keap1* reduced the higher protein abundances of fibrosis markers and apoptosis‐related indices, as well as antioxidant proteins NRF2 and HO‐1 in Ang II‐exposed NRK cells; this protective effect was not further enhanced by *Cirbp*‐OE (Figure 8H,I), but reversed by si‐*Cirbp* (Figure 8J,K). On the mRNA level, si‐*Keap1* promoted the activation of the antioxidant gene *Hmox1* and *Nqo1*, and *Cirbp*‐OE did not further enhance (Supporting Information S1: Figure S7E), while si‐*Cirbp* reversed this effect (Supporting Information S1: Figure S7F). DCFH‐DA and DHE staining revealed that si‐*Keap1* exerted an inhibitory effect on oxidative stress elicited by Ang II, whereas this effect was not further amplified by *Cirbp*‐OE (Supporting Information S1: Figure S7G,H) but reversed by si‐*Cirbp* (Supporting Information S1: Figure S7I,J). These results suggested that *Keap1* deficiency exerted anti‐fibrotic and anti‐apoptotic effects in Ang II‐treated cells through the NRF2 activation.

### 3.9 CIRBP Protects Against Ang II‐Induced Renal Damage via the KEAP1‐NRF2 Pathway

We examined whether CIRBP regulates KEAP1 expression and thereby affects downstream NRF2 signaling and renal injury. We evaluated the effects of the pharmacological NRF2 agonist RA839 together with those of the NRF2 inhibitor ML385 on renal apoptosis, fibrosis, and oxidative stress. RA839 reduced the increased protein levels of the fibrosis markers COL Ia1 and α‐SMA and the apoptotic markers BCL‐2/BAX and cleaved caspase 3/caspase 3, as well as NRF2 target HO‐1, in Ang II‐treated NRK cells. Additionally, RA839 rescued the harmful effects of *Cirbp* knockdown combined with Ang II (Figure 9A,B and Supporting Information S1: Figure S8A,B). RA839 inhibited the Ang II‐induced transcriptional repression of NRF2‐targeted antioxidant genes *Hmox1* and *Nqo1*, and alleviated cellular damage caused by si‐*Cirbp* (Supporting Information S1: Figure S8E). In contrast, ML385 reversed the protection conferred by *Cirbp*‐OE against Ang II‐induced fibrotic injury and apoptosis and aggravated the injury under physiological conditions (Figure 9C,D and Supporting Information S1: Figure S8C,D). The downstream antioxidant genes had the same results at the mRNA level (Supporting Information S1: Figure S8F). DCFH‐DA staining showed that RA839 produced an inhibitory effect against Ang II‐induced oxidative stress, whereas this effect was reversed by si‐*Cirbp* (Figure 9E,F). DHE staining showed similar results (Figure 9G,H). Similarly, both probes demonstrated that ML385 elevated basal ROS levels in NRK cells and reversed the protective action of *Cirbp*‐OE against Ang II‐associated renal oxidative stress (Figure 9I–L). These results indicated that *Cirbp* alleviated renal oxidative stress and renal injury through the KEAP1‐NRF2 pathway.

**FIGURE 9:**
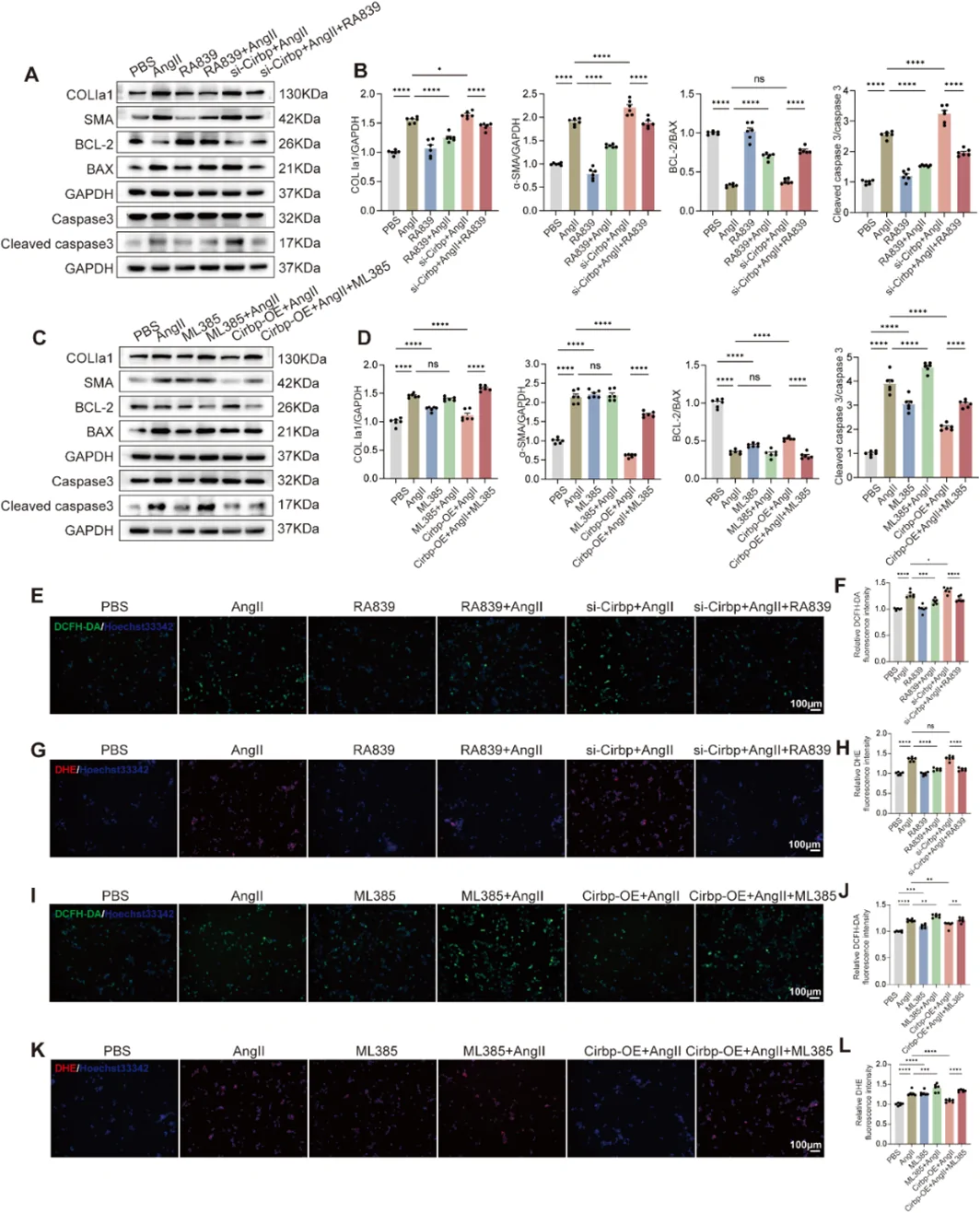
CIRBP relieves Ang II‐evoked renal damage through the KEAP1‐NRF2 axis. (A, B) Western blotting assessment of COL Ia1, α‐SMA, BCL‐2/BAX, cleaved caspase 3/caspase 3 abundance in NRK with si‐*Cirbp* and/or RA839 after PBS or Ang II stimulation (*n* = 6). (C, D) Corresponding Western blotting quantification in Cirbp‐OE NRK cells treated with ML385 (*n* = 6). (E, F) DCFH‐DA detection of ROS in si‐*Cirbp* cells treated with RA839 under PBS or Ang II conditions (*n* = 6). (G, H) DHE labeling of ROS within NRK cultures with si‐*Cirbp* and/or RA839 after PBS or Ang II stimulation (*n* = 6). (I, J) DCFH‐DA staining for ROS in NRK cells with *Cirbp*‐OE and/or ML385 after PBS or Ang II stimulation (*n* = 6). (K, L) DHE‐based ROS detection in Cirbp‐OE NRK cells with or without ML385 (*n* = 6).

## 4 Discussion

In this study, *Cirbp* was discovered as a novel regulatory factor in Ang II‐mediated renal injury, and its expression was downregulated in mice subjected to chronic pressure overload induced by Ang II stimulation. In mouse models, *Cirbp* deficiency aggravated, whereas *Cirbp* overexpression ameliorated Ang II‐induced renal dysfunction, fibrosis, apoptosis, inflammation and oxidative stress. Mechanistically, the renoprotective effect of CIRBP rose out of the alleviation on the oxidative stress response via activating the KEAP1‐NRF2 antioxidant‐response axis. These findings advocate the potentiality of *Cirbp* or NRF2 agonists as targets for preventing the onset and progression of hypertensive nephropathy.

Angiotensin receptors, widely expressed in the vasculature and glomeruli, can drive progressive renal injury through multiple pathways [18]. Ang II contributes to hypertensive nephropathy by constricting both afferent and efferent arterioles. As a profibrotic cytokine, Ang II promotes fibrotic remodeling by inducing transforming growth factor‐β (*TGF‐β*) expression [19]. In addition, Ang II both activates NADPH oxidases [20], and disrupts mitochondrial electron transport via the AT1 receptor to trigger intracellular oxidative stress cascades [21], thereby making it a major contributor to secondary renal injury. This dysregulation of the redox state can induce podocyte apoptosis, cytoskeletal disintegration, and hypertensive renal injury [22]. ROS accumulate to initiate sterile inflammation through activation of NF‐κB [5], creating a permissive environment for ferroptosis [23]. Together, these findings highlight the interplay between redox homeostasis, inflammation, and other forms of injury engaged in hypertensive nephropathy. A clearer insight into the drivers of oxidative stress activation could support the development of targeted therapies to prevent or alleviate renal injury.

Recent studies suggest that *Cirbp* may exert distinct, and in some contexts even opposing, effects in iodinated contrast agent‐induced acute kidney injury, depending on its site of expression and the nature of the stimulus [24]. Previous research has shown that the extracellular secretion of *Cirbp* heightens inflammatory cascades in some models of acute injury [25, 26]. In kidney transplant recipients, higher serum *Cirbp* is related to higher plasma creatinine at 6 months after transplantation [27], suggesting that *Cirbp* could serve as a biomarker in post‐transplant kidney function assessment. In contrast, intracellular *Cirbp* acts as a cytoprotective factor. *Cirbp* regulates cellular adaptation to environmental stressors, including low temperature [28], hypoxia [6], and ultraviolet radiation [29], and ameliorates the disturbances in energy metabolism [30]. In glioblastoma cells undergoing erastin‐induced ferroptosis, *Cirbp* is differentially expressed, suggesting its involvement in cell death [31]. A high protein level of *Cirbp* expression alleviates apoptosis caused by AKT pathway activation, and enhances mitophagy in yak cumulus cells by stimulating the AMPK/mTOR pathway [32]. In addition, *Cirbp*‐containing toxic exon isoforms can reduce the intracellular bacterial burden [33]. In tissues demanding for a high metabolic energy, the hypothermic cardioprotective effect in hearts from young donors is attenuated in *Cirbp*‐KO rats, because of reduced dihydroorotate dehydrogenase‐mediated ubiquinone production and subsequent ferroptosis [34]. *Cirbp* stabilizes *Slc7a11* and *Gpx4* to limit ferroptosis and protect against doxorubicin‐induced cardiotoxicity [8]. *Cirbp* also shortens the time to hypothermia and alleviates intrinsic apoptosis and NLRP3 inflammatory responses in the liver [10]. In this study, *Cirbp* expression was downregulated in Ang II‐induced kidneys, and *Cirbp* overexpression attenuated apoptotic and inflammatory cascades by reducing renal oxidative stress.

*Nrf2*, a ubiquitous master transcription factor, can inhibit oxidative stress [11]. In murine models with renal ischemia‐reperfusion injury (IRI), the activation of the antioxidant transcription factor *Nrf2* in renal tubules effectively alleviates tubular injury and inhibits interstitial fibrosis [35]. Zahran et al. have reported that Rhus coriaria protects against renal injury caused by MTX‐induced nephrotoxicity by reducing *Keap1* expression and increasing the expression of *Hmox1* and *Nrf2* [36]. AX‐SLN curbs breast cancer progression through the same mechanism [37]. Our study further showed that CIRBP executes its renoprotective effects via the KEAP1‐NRF2 axis, and that a NRF2 agonist alleviates Ang II‐induced renal injury [38]. Previous studies have suggested a synergistic relationship between *Cirbp* and the KEAP1‐NRF2 pathway [39], but their interaction remains incompletely defined. Here, we characterized an interaction between Cirbp and Keap1 through qPCR, Western blotting, CO‐IP, colocalization analysis, and functional rescue experiments. The data demonstrated that CIRBP bound to KEAP1 and promoted its ubiquitination‐mediated degradation. As a highly conserved stress‐responsive RNA‐binding protein, Cirbp may respond to microenvironmental changes by altering the conformation of docking proteins at specific sites [33].

These findings are meaningful in the management of hypertensive nephropathy. Existing therapies aiming at the renin‐angiotensin‐aldosterone system (RAAS) can delay, but not prevent, the progression of chronic kidney disease [40]. Interventions that enhance antioxidant capacity therefore represent a direct adjunctive strategy. Our evidence suggests that *Cirbp* may serve as a therapeutic target, though warranting further evaluation. Ang II stimulation suppresses nuclear NRF2 localization and weakens the expression of downstream antioxidant proteins. Strategies aimed at specifically upregulating intracellular *Cirbp* expression or activating its downstream effectors may help restore cellular redox homeostasis and prevent Ang II‐induced cytotoxicity.

Several limitations should be noted in this study. First, it relied mainly on systemic overexpression and knockout models. Future work should resolve the roles across different cell types using renal tubule‐specific or podocyte‐specific *Cirbp* knockout models. Second, although we observed that the CIRBP protein regulates the KEAP1‐NRF2 pathway, the direct binding motif remains to be verified. Finally, we validated the effects of downstream agonists only at the cellular level, and additional studies should be conducted based on animal models.

Our study identified CIRBP acts on the Ang II‐driven renal injury through the CIRBP‐KEAP1‐NRF2 signaling axis, and may promise as a potential therapeutic target for kidney damage induced by Ang II and redox imbalance.

## Author Contributions

**Yue Zhang:** conceptualization, experiments, writing – original draft. **Qilin Li:** conceptualization, experiments, writing – original draft. **Man Luo:** conceptualization, experiments, writing – original draft. **Dongxu Hua:** methodology. **Yanhui Sheng:** conceptualization, formal analysis, writing – review and editing, supervision, funding acquisition. **Lei Wang:** conceptualization, writing – review and editing, supervision. **Peng Li:** conceptualization, formal analysis, writing – review and editing, supervision, funding acquisition.

## Ethics Statement

All animal procedures were approved by the Ethics Committee at Nanjing Medical University (2101039).

## Conflicts of Interest

The authors declare no conflicts of interest.

## Supporting information


Supporting Information S1

## Data Availability

All data supporting the conclusion have been included within the article and/or the supplementary materials. The corresponding author can provide additional data on request.
