# Supplementary material for: Cold‐Inducible RNA‐Binding Protein Alleviates Hypertensive Renal Injury Through the KEAP1‐NRF2 Pathway
Source: Smart Med. 2026 Nov 7;5(6):e70062. doi: 10.1002/smmd.70062 (PMC13744398; doi:10.1002/smmd.70062)
Supplement: Supplementary file 1 [file SMMD-5-e70062-s001.pdf]

Supplementary Table 1: The primer sequence used for quantitative PC

Gene	Species	Forward primer	Reverse primer
Cirbp	Mice	AGACTACTATGCCAGCCGGA	GGCATCCTTGGAGCTAGACTT
Ngal	Mice	ACTCTGGGAAATATGCACAGGTA	TGGCGAACTGGTTGTAGTCC
Col-I	Mice	GCTCCTCTTAGGGGCCACT	CCACGTCTCACCATTGGGG
α-sma	Mice	GTCCCAGACATCAGGGAGTAA	TCGGATACTTCAGCGTCAGGA
Bcl-2	Mice	ATGCCTTTGTGGAACTATATGGC	GGTATGCACCCAGAGTGATGC
Bax	Mice	TGAAGACAGGGGCCTTTTTG	AATTCGCCGGAGACACTCG
Il-6	Mice	CCAAGAGGTGAGTGCTTCCC	CTGTTGTTCAGACTCTCTCCCT
Tnf-α	Mice	GACGTGGAACTGGCAGAAGAG	TTGGTGGTTTGTGAGTGTGAG
Gapdh	Mice	AGGTCGGTGTGAACGGATTTG	TGTAGACCATGTAGTTGAGGTCA
Keap1	Mice	CCTGTGCCTCTATGAGCGTG	CGCTCTGGCTCATATCTCTCC
Hmox1	Mice	GCCACCAAGGAGGTACACAT	AGGAAGCCATCACCAGCTTAAA
Nqo1	Mice	TCTCTGGCCGATTCAGAGTG	CCAGACGGTTTCCAGACGTT
Cirbp	Rat	GGTGGTGGTAAAGGACAGGG	GCCTCCCATCCACAGACTTC
Ngal	Rat	AAGTCACCCTGTACGGAAGAAC	CGGTGGGAACAGAGAAAACGA
Col-I	Rat	AGAGGCATAAAGGGTCATCGTG	AGACCGTTGAGTCCATCTTTGC
α-sma	Rat	AACCGGGAGAAAATGACCCA	AGTCCAGCACAATACCAGTTG
Bcl-2	Rat	CATGTGTGTGGAGAGCGTCA	TAGTTCCACAAAGGCATCCCAG
Bax	Rat	GCCTTTTTGCTACAGGGTTTCAT	TTGTTGTCCAGTTCATCGCCA
Il-6	Rat	CCAGTTGCCTTCTTGGGACT	CTGGTCTGTTGTGGGTGGTA
Tnf-α	Rat	ATGGGCTCCCTCTCATCAGT	GCTTGGTGGTTTGCTACGAC
Gapdh	Rat	GGCACAGTCAAGGCTGAGAATG	ATGGTGGTGAAGACGCCAGTA
Keap1	Rat	CCATCGGCATCGCCAACTTC	GAAGAACTCCTCCTCCCCGA
Hmox1	Rat	GTCCCAGGATTTGTCCGAGG	GGAGGCCATCACCAGCTTAAA
Nqo1	Rat	CAGAAACGACATCACAGGGGA	GGCCTTCCTTATACGCCAGA

Supplementary Table 2: Differentially expressed genes in the three datasets

P value < 0.05				FDR < 0.05
Adam10	*Spc25*	*Me2*	*Gcdh*	*Calu*
Agfg1	*Stag1*	*Mllt3*	*Grhpr*	*Copb1*
Ascc3	*Stam*	*Mtf2*	*Grpel1*	*Dhx15*
Calu	*Stk39*	*Mtmr6*	*Hap1*	*Dnajc10*
Cast	*Sub1*	*Mycbp*	*Hsf4*	*Kpna2*
Cenpq	*Thoc7*	*Nemf*	*Klhdc4*	*Mtf2*
Ckap5	*Tmem97*	*Nipa2*	*Ndor1*	*Pibf1*
Cnot2	*Tmx1*	*Npc1*	*Ndufb11*	*Ppwd1*
Copb1	*Tram1*	*Pdcd10*	*Ndufb7*	*Stag1*
Cops2	*Trim24*	*Pdia3*	*Neu3*	*Stam*
Cp	*Trim37*	*Phf14*	*Ogfod2*	*Uba6*
Cyfip1	*Uba6*	*Pibf1*	*Plekhh3*	*Zcchc10*
Dhx15	*Vps35*	*Pkia*	*Pmvk*	*Cirbp*
Dnajc10	*Vps41*	*Plcb4*	*Polm*	*Clpp*
Drosha	*Ythdf2*	*Pls3*	*Qdpr*	*Erf*
Dyrk2	*Zcchc10*	*Ppt1*	*Rab20*	*Fkbp8*
Ecd	*Acads*	*Ppwd1*	*Ring1*	*Grhpr*
Ermp1	*Bin1*	*Psip1*	*Spsb3*	*Tex264*
Fbxo11	*Cirbp*	*Psmc6*	*Tbkbp1*	*Tfeb*
Fn1	*Clpp*	*Psme4*	*Tex264*	
Focad	*Dctn3*	*Pttg1*	*Tfeb*	
Gabpa	*Dexi*	*Rnf139*	*Tmem134*	
Il33	*Dnpep*	*Rpe*	*Tspan7*	
Kidins220	*Ephx1*	*Rpn2*	*Tspan9*	
Kpna2	*Erf*	*Rps19*	*Txnrd2*	
Kras	*Fgfr3*	*Rrm1*	*Ubxn6*	
Mapk6	*Fkbp8*	*Smap1*	*Uros*	
Mapre1	*Fxr2*	*Snx16*	*Vamp2*	

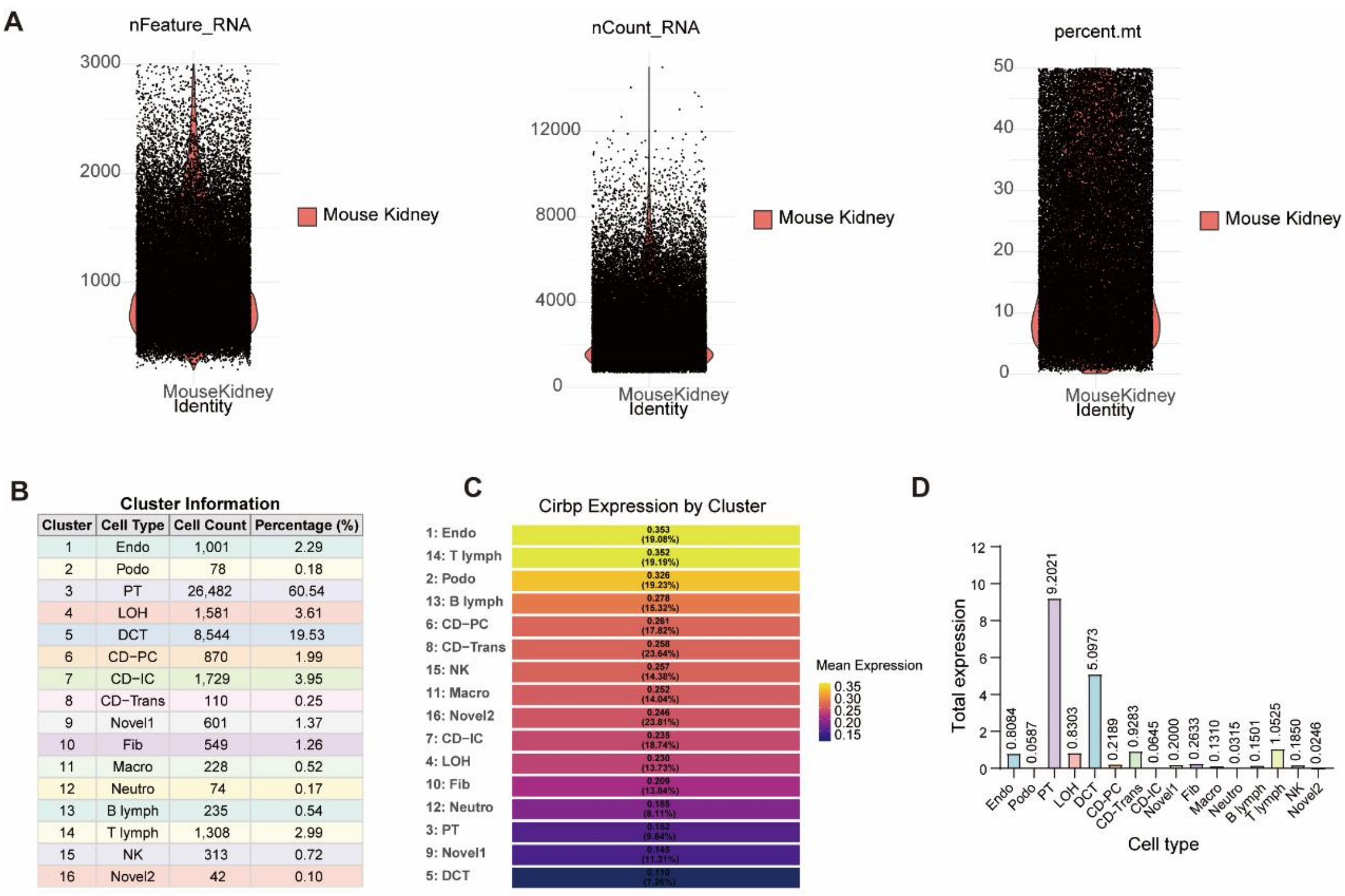

Cluster Information

Cluster	Cell Type	Cell Count	Percentage (%)
1	Endo	1,001	2.29
2	Podo	78	0.18
3	PT	26,482	60.54
4	LOH	1,581	3.61
5	DCT	8,544	19.53
6	CD−PC	870	1.99
7	CD−IC	1,729	3.95
8	CD−Trans	110	0.25
9	Novel1	601	1.37
10	Fib	549	1.26
11	Macro	228	0.52
12	Neutro	74	0.17
13	B lymph	235	0.54
14	T lymph	1,308	2.99
15	NK	313	0.72
16	Novel2	42	0.10

Supplemental Fig. 1 Single-cell transcriptomic analysis of mouse kidney samples in GSE107585. A, Quality control of nFeature, nCount and mitochondria percentage. B, Cell count and percentage of 16 distinct cell types displayed on a UMAP plot. Endo, endothelial, vascular, and descending loop of Henle cells; Podo, podocytes; PT, proximal tubules; LOH, ascending loop of Henle; DCT, distal convoluted tubules; CD-PC, collecting duct principal cells; CD-IC, collecting duct intercalated cells; CD-Trans, collecting duct transitional cells; Fib, fibroblasts; Macro, macrophages; Neutro, neutrophils; NK, natural killer cells. C, The average *Cirbp* expression level and the proportion of *Cirbp*-positive cells in each renal cell type. D, Total *Cirbp* expression level in each renal cell type.

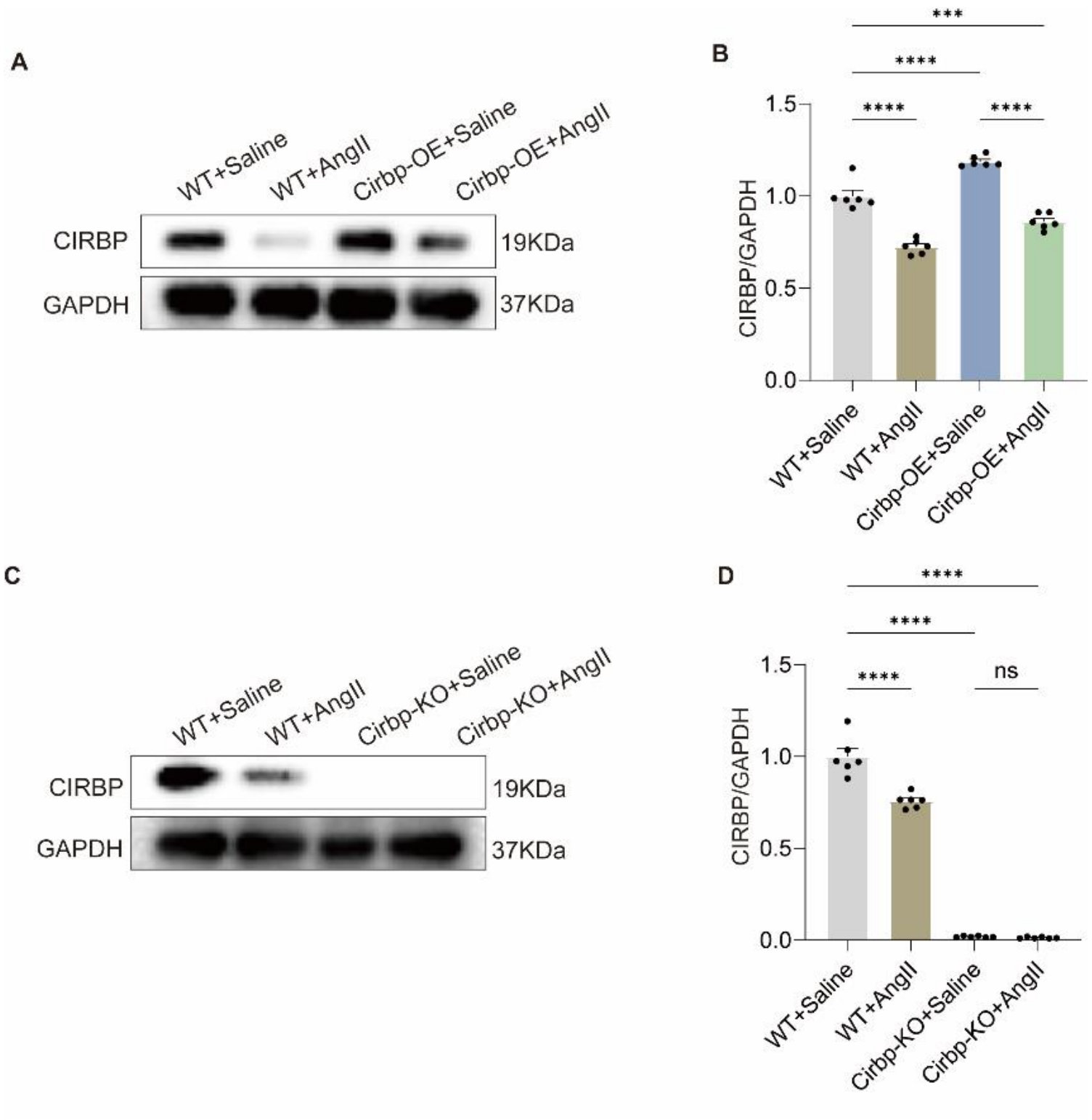

Supplemental Fig. 2 Verification of CIRBP overexpression and knockout efficiency in mice. A and B, Western blot of CIRBP protein level in kidneys from WT and *Cirbp*-OE mice after Saline or Ang II infusion (n=6). C and D, Western blot of CIRBP protein level in kidneys from WT and *Cirbp*-KO mice after Saline or Ang II infusion (n=6). GAPDH was used as a loading control. Data are expressed as the mean ± SEM. Two-way ANOVA and Tukey's Multiple Comparison Test were performed. ns, not significant, * $P < 0.05$, ** $P < 0.01$, *** $P < 0.001$, **** $P < 0.0001$.

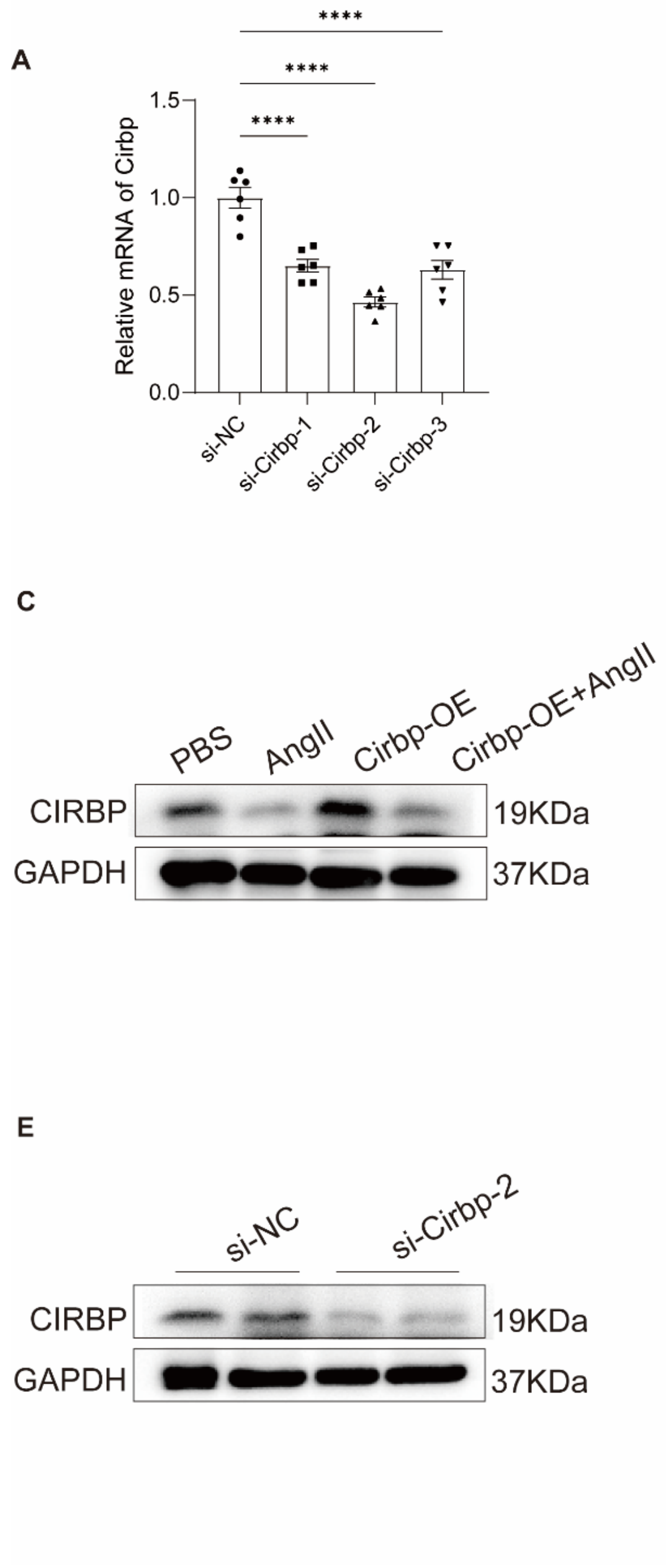

Supplemental Fig. 3 Verification of siRNA and plasmid efficiency in NRK-52E cells. A, qRT–PCR analysis of *Cirbp* mRNA level in NRK-52E with si-*Cirbp* (n=6). B, qRT–PCR analysis of *Cirbp* mRNA level in NRK-52E with *Cirbp*-OE (n=6). C and D, Western blot of CIRBP protein level in NRK-52E with *Cirbp*-OE after PBS or Ang II stimulation (n=6). E and F, Western blot of CIRBP protein level in NRK-52E with si-*Cirbp*-2 (n=6). GAPDH was used as a loading control. Data are expressed as the mean ± SEM. Unpaired t test was performed for A, B and F, Two-way ANOVA and Tukey's Multiple Comparison Test were performed for D. ns, not significant, * $P < 0.05$, ** $P < 0.01$, *** $P < 0.001$, **** $P < 0.0001$.

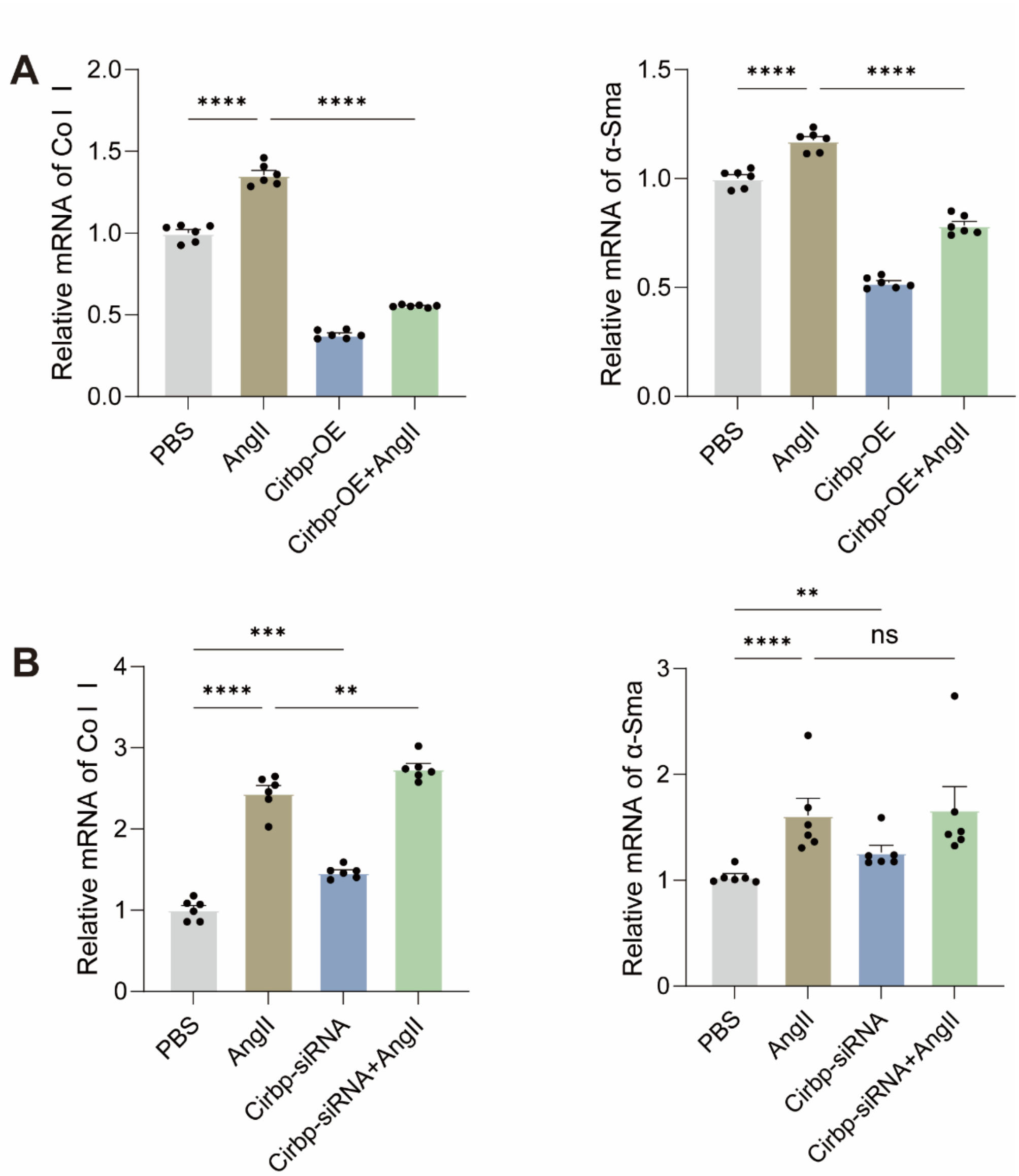

Supplemental Fig. 4 *Cirbp* alleviated renal fibrosis in vitro. A, mRNA expression of fibroblast activation-related marker genes (*Col I* and *α-sma*) in NRK with *Cirbp*-OE after PBS or Ang II stimulation (n = 6). B, mRNA expression of *Col I* and *α-sma* in NRK with si-*Cirbp* after PBS or Ang II stimulation (n = 6). The loading control was detected to be *Gapdh*. The results are expressed as the mean ± SEM. Two-way ANOVA and Tukey's Multiple Comparison Test were performed. ns, not significant, **P*<0.05, ***P*<0.01, ****P*<0.001, *****P*<0.0001.

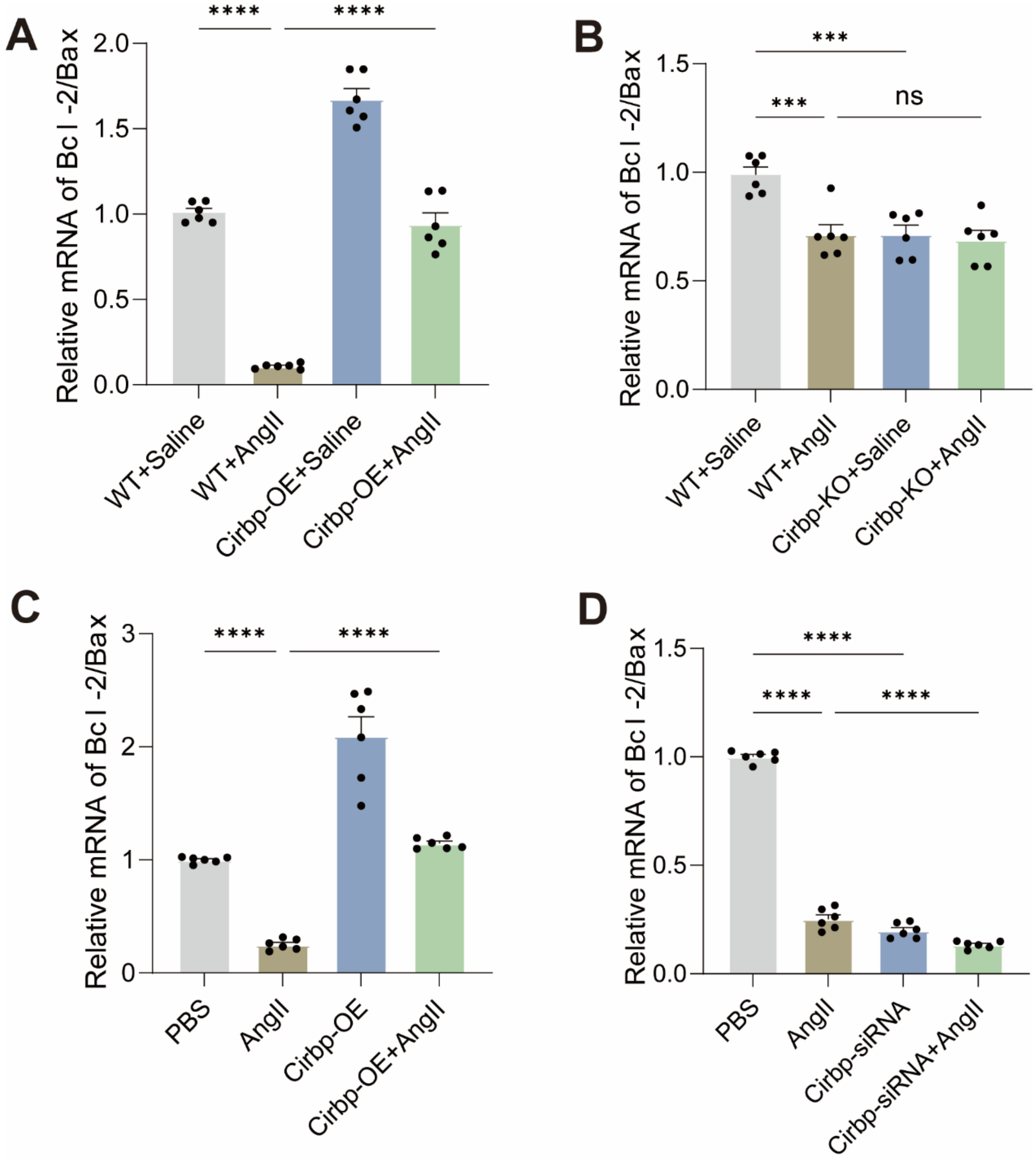

Supplemental Fig. 5 *Cirbp* alleviated renal apoptosis in vivo and vitro. A, mRNA expression of apoptosis-related marker genes (Bcl-2 / Bax) in kidneys from WT and *Cirbp*-OE or *Cirbp*-KO mice after Saline or Ang II infusion (n = 6). B, mRNA expression of Bcl-2 / Bax in NRK with *Cirbp*-OE or si-*Cirbp* after PBS or Ang II stimulation (n = 6). The loading control was detected to be *Gapdh*. The results are expressed as the mean ± SEM. Two-way ANOVA and Tukey's Multiple Comparison Test were performed. ns, not significant, **P*<0.05, ***P*<0.01, ****P*<0.001, *****P*<0.0001.

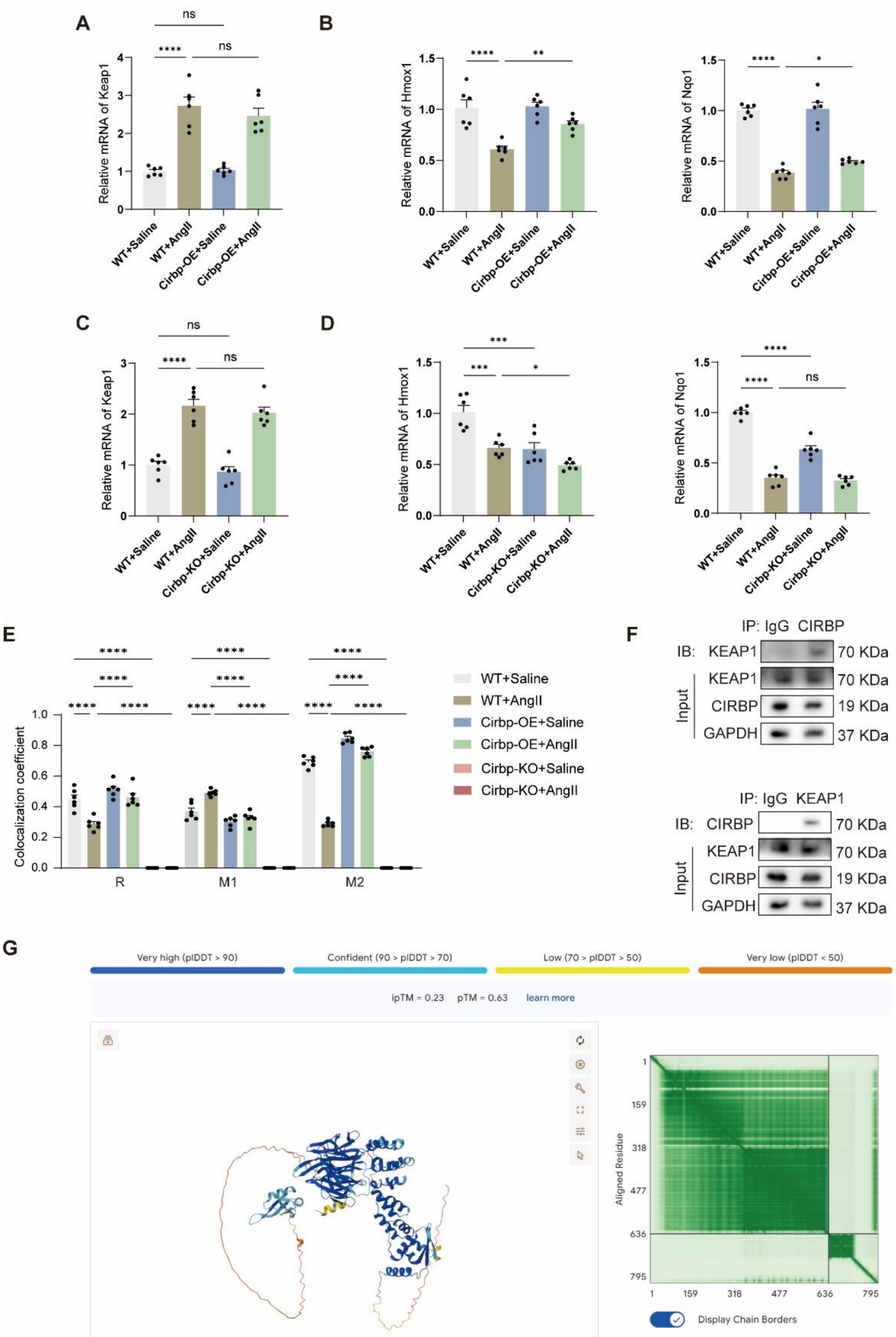

Supplemental Fig. 6 Cirbp decreases Keap1 expression and interactions between CIRBP and KEAP1. A and B, mRNA expression of *Keap1*, *Hmox1* and *Nqo1* in kidneys from WT and *Cirbp*-OE mice after Saline or Ang II infusion (n = 6). C and D, mRNA expression of *Keap1*, *Hmox1* and *Nqo1* in kidneys from WT and *Cirbp*-KO mice after Saline or Ang II infusion (n = 6). E, Pearson's correlation coefficient and Manders' overlap coefficients M1 (fraction of

CIRBP overlapping KEAP1) and M2 coefficient (fraction of KEAP1 overlapping CIRBP) of immunofluorescence of CIRBP and KEAP1. F, Endogenous and reciprocal Co-IP and Western blotting of CIRBP and KEAP1 in the NRK-52E cells. G, AlphaFold 3 predicted the binding potential of KEAP1 and CIRBP. The results are expressed as the mean ± SEM. Two-way ANOVA and Tukey's Multiple Comparison Test were performed. ns, not significant, *$P<0.05$, **$P<0.01$, ***$P<0.001$, ****$P<0.0001$.

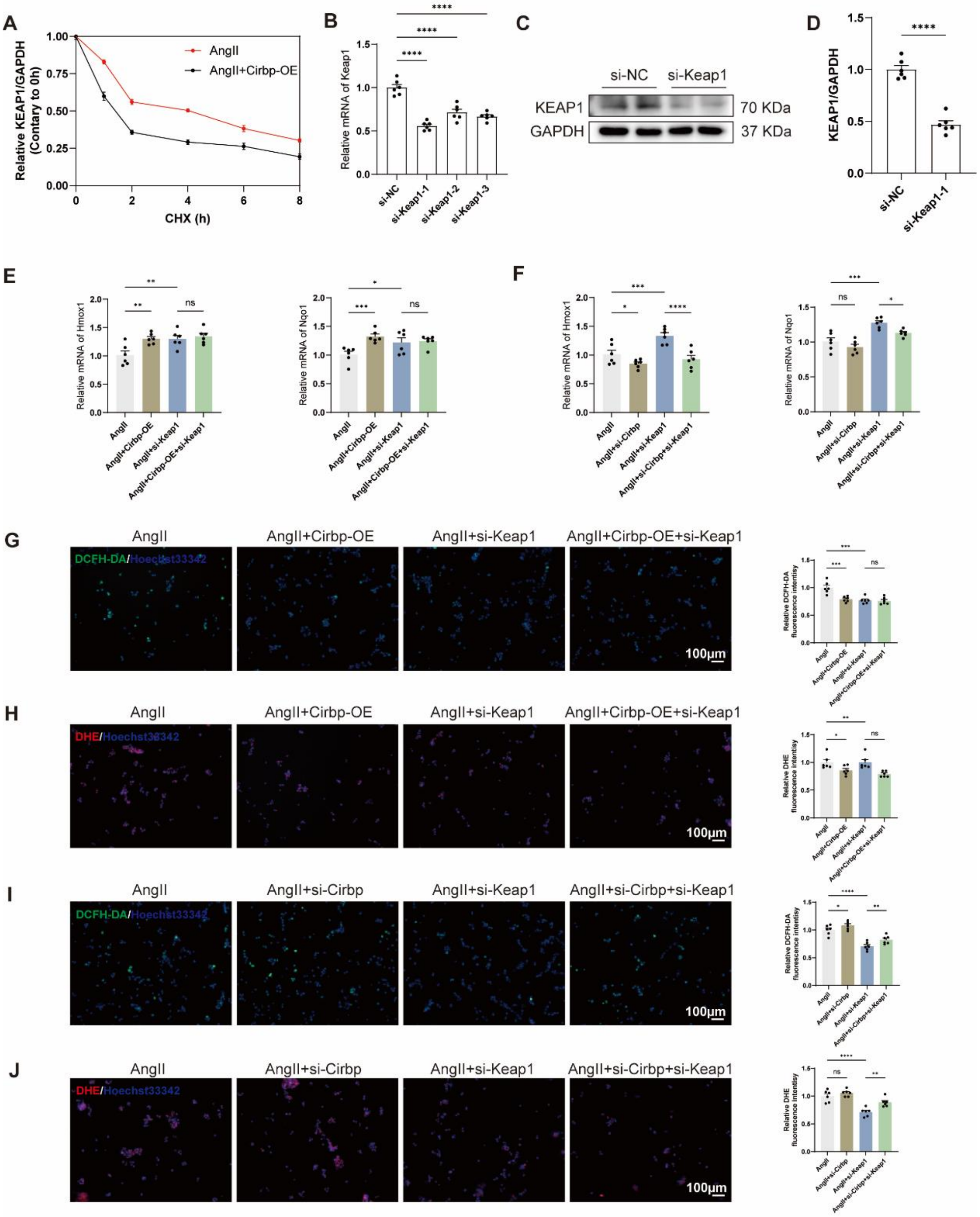

Supplemental Fig. 7 Function reversal experiment of Keap1 knock down. A, Cycloheximide (CHX) chase assay showing KEAP1 protein stability in *Cirbp*-OE NRK-52E cells treated with cycloheximide (0,1,2,4,6,8 h), relative to CHX 0h (n=6). B, qRT–PCR analysis of *Keap1* mRNA level in NRK-52E with si-*Keap1* (n=6). C and D, Western blot of KEAP1 protein level in NRK-52E with si-*Keap1*-1 (n=6). GAPDH was used as a loading control. E, qPCR analysis of *Hmox1*, and *Nqo1* mRNA levels in NRK-52E with Cirbp-OE or si-Keap1 after saline or Ang II infusion (n = 6). F, qPCR analysis of *Hmox1*, and *Nqo1* mRNA

levels in NRK-52E with si-*Cirbp* or si-*Keap1* after saline or Ang II infusion (n = 6). G, DCFH-DA staining of ROS in NRK cells with si-*Keap1* and/or *Cirbp*-OE after PBS or Ang II stimulation (n = 6; scale bar = 100 μm). H, DHE staining of ROS in NRK cells with si-*Keap1* and/or *Cirbp*-OE after PBS or Ang II stimulation (n = 6; scale bar = 100 μm). I, DCFH-DA staining of ROS in NRK cells with si-*Keap1* and/or si-*Cirbp* after PBS or Ang II stimulation (n = 6; scale bar = 100 μm). J, DHE staining of ROS in NRK cells with si-*Keap1* and/or si-*Cirbp* after PBS or Ang II stimulation (n = 6; scale bar = 100 μm). The results are expressed as the mean ± SEM. Two-way ANOVA was performed on B; Unpaired t test was performed on D; Two-way ANOVA and Tukey's Multiple Comparison Test were performed on others. ns, not significant, **P*<0.05, ***P*<0.01, ****P*<0.001, *****P*<0.0001.

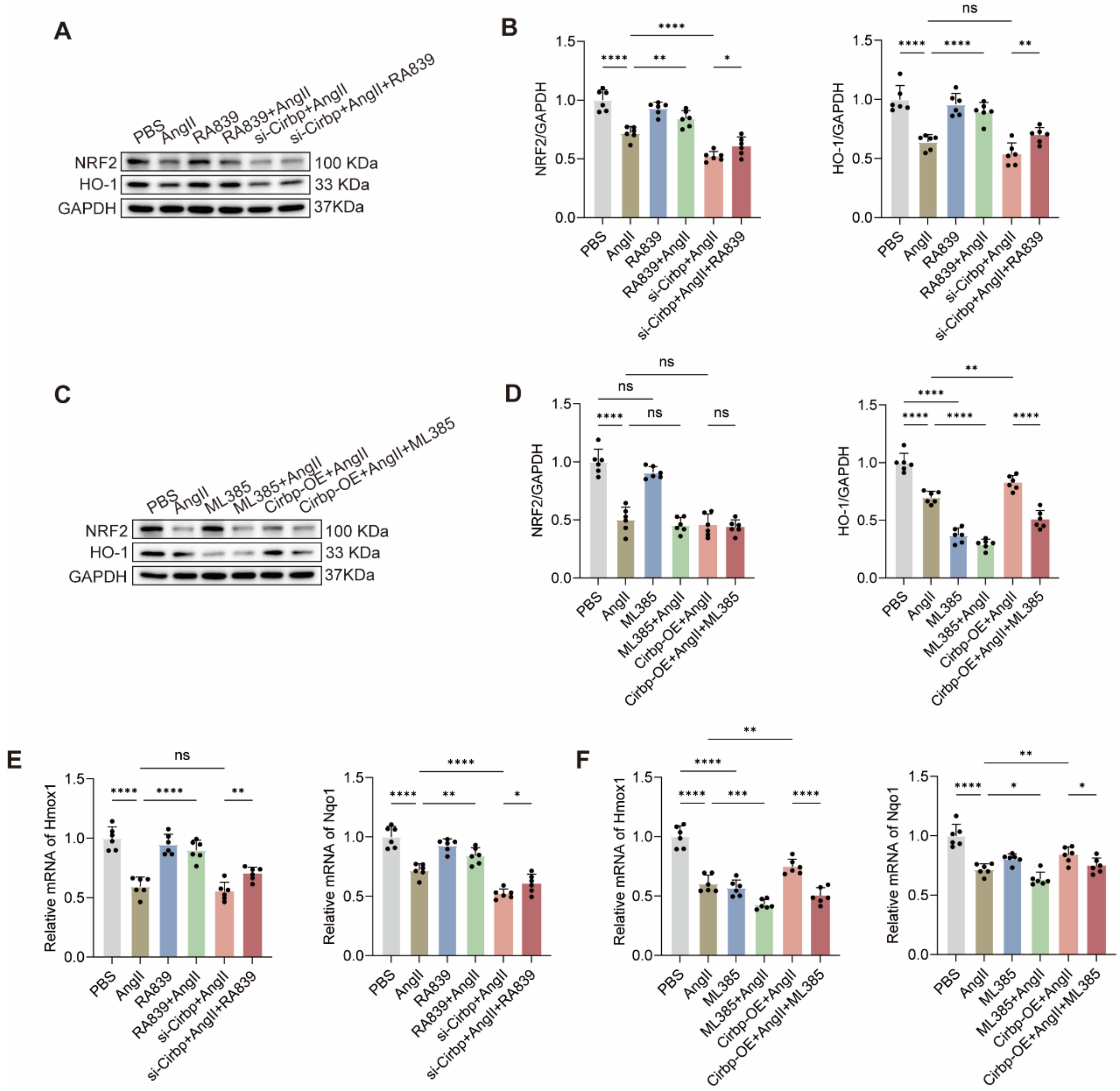

Supplemental Fig. 8 mRNA of antioxidant genes with RA839 or ML385 intervention. A, A and B, Western blotting analysis of NRF2 and HO-1 protein levels in NRK cells with si-*Cirbp* and/or RA839 after PBS or Ang II stimulation (n = 6). C and D, Western blotting analysis of NRF2 and HO-1 protein levels in NRK cells with *Cirbp*-OE and/or ML385 after PBS or Ang II stimulation (n = 6). E, qPCR analysis of *Hmox1* and *Nqo1* mRNA levels in NRK cells with si-*Cirbp* and/or RA839 after PBS or Ang II stimulation (n = 6). F, qPCR analysis of *Hmox1* and *Nqo1* mRNA levels in NRK cells with *Cirbp*-OE and/or ML385 after PBS or Ang II stimulation (n = 6). The results are expressed as the mean ± SEM. Two-way ANOVA and Tukey's Multiple Comparison Test were performed. ns, not significant, **P*<0.05, ***P*<0.01, ****P*<0.001, *****P*<0.0001.
